# Rescue of GM3 synthase deficiency by spatially controlled, rAAV-mediated *ST3GAL5* delivery

**DOI:** 10.1172/jci.insight.168688

**Published:** 2023-05-08

**Authors:** Huiya Yang, Robert H. Brown, Dan Wang, Kevin A. Strauss, Guangping Gao

**Affiliations:** 1 Horae Gene Therapy Center,; 2 Department of Neurology,; 3 Li Weibo Institute for Rare Diseases Research, and; 4 RNA Therapeutics Institute, University of Massachusetts Chan Medical School, Worcester, Massachusetts, USA.; 5 Clinic for Special Children, Strasburg, Pennsylvania, USA.; 6 Department of Molecular, Cell and Cancer Biology, and; 7 Department of Microbiology and Physiological Systems, University of Massachusetts Chan Medical School, Worcester, Massachusetts, USA.

**Keywords:** Neuroscience, Therapeutics, Gene therapy, Neurological disorders

## Abstract

GM3 synthase deficiency (GM3SD) is an infantile-onset epileptic encephalopathy syndrome caused by biallelic loss-of-function mutations in *ST3GAL5*. Loss of ST3GAL5 activity in humans results in systemic ganglioside deficiency and severe neurological impairment. No disease-modifying treatment is currently available. Certain recombinant adeno-associated viruses (rAAVs) can cross the blood-brain barrier to induce widespread, long-term gene expression in the CNS and represent a promising therapeutic strategy. Here, we show that a first-generation rAAV-*ST3GAL5* replacement vector using a ubiquitous promoter restored tissue ST3GAL5 expression and normalized cerebral gangliosides in patient-derived induced pluripotent stem cell neurons and brain tissue from *St3gal5*-KO mice but caused fatal hepatotoxicity when administered systemically. In contrast, a second-generation vector optimized for CNS-restricted ST3GAL5 expression, administered by either the intracerebroventricular or i.v. route at P1, allowed for safe and effective rescue of lethality and behavior impairment in symptomatic GM3SD mice up to a year. These results support further clinical development of *ST3GAL5* gene therapy.

## Graphical abstract

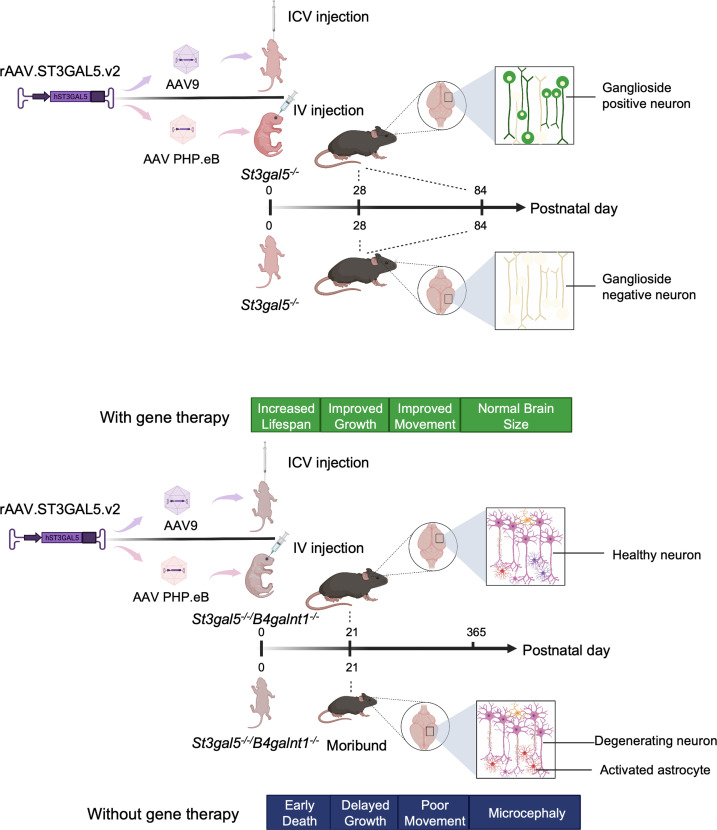

## Introduction

**Figure 1:**
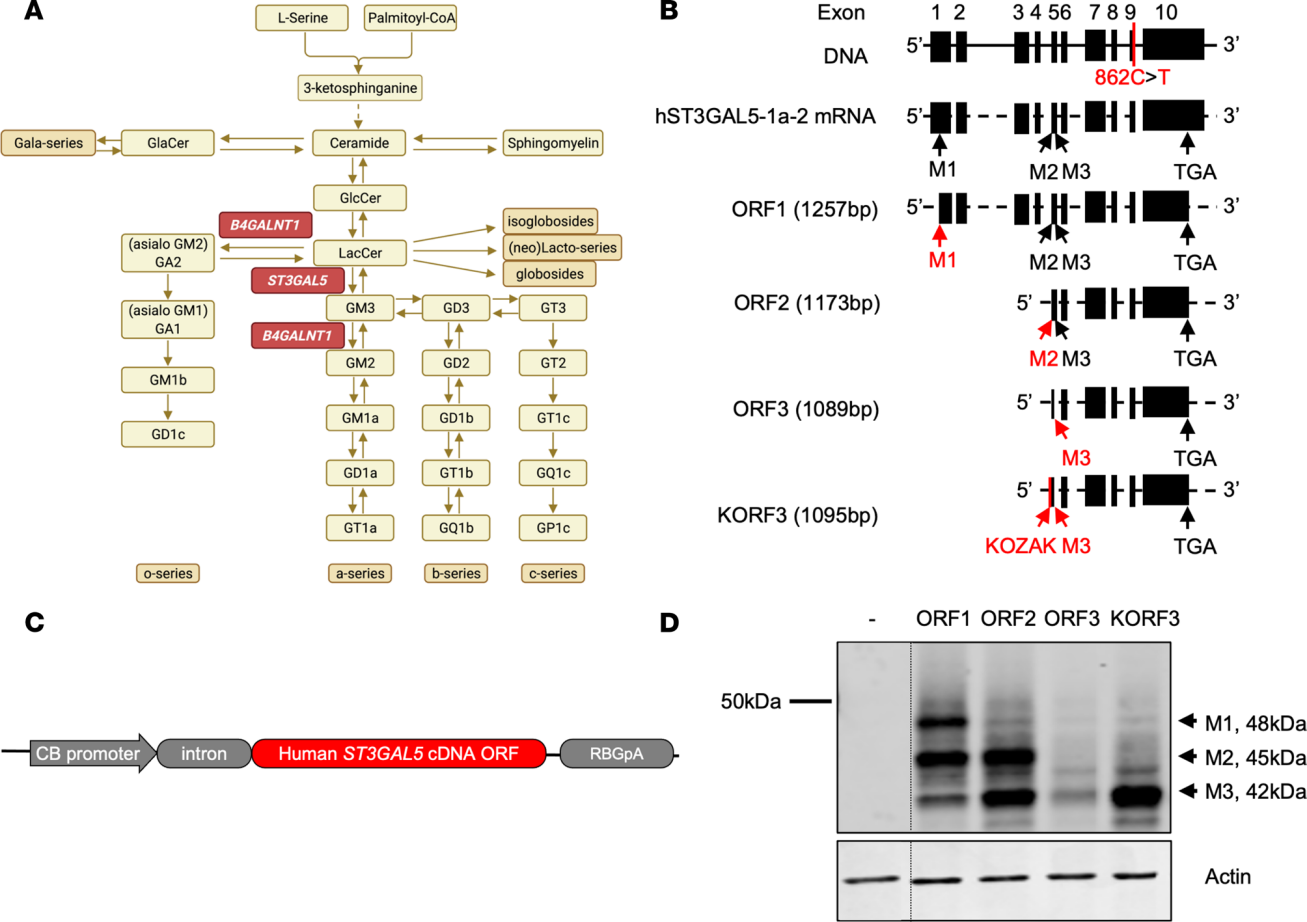
GM3SD is caused by loss-of-function mutation of *ST3GAL5*. (**A**) Schematic showing de novo gangliosides’ synthesis pathway. ST3GAL5 uses LacCer as a substrate to synthesize GM3, the precursor of all other gangliosides. B4GALNT1 is another key enzyme to catalyze the complex gangliosides’ formation. Loss-of-function mutations in *ST3GAL5* and *B4GALNT1* cause GM3SD and hereditary spastic paraplegia type 26, respectively. (**B**) Schematic of the human *ST3GAL5* DNA genome and the most abundant mRNA isoform noted in National Center for Biotechnology Information (NM_003896). M1, M2, and M3 represent the 3 initiating start codons for methionine. Stop codon TGA is at exon 10, and Amish mutation (p.862C>T) is located at exon 9. cDNA initiating from M1 (ORF1), M2 (ORF2), M3 (ORF3), and Kozak+M3 (KORF3) is of 1257 bp, 1173 bp, 1089 bp, and 1095 bp, respectively. Black boxes represent exons; black lines represent introns; dashed black lines represent spliced introns. (**C**) A construct expressing ubiquitous human *ST3GAL5* ORF is shown. (**D**) Representative Western blot images of ST3GAL5 protein expression via different ORF transfections in HeLa cells. The thin black dividing line indicates splicing of noncontiguous lanes of the same blot.

*ST3GAL5* encodes GM3 synthase (ST3GAL5; also called GM3S and SIAT9), the rate-limiting enzyme for production of all a- and b-series gangliosides normally enriched in mammalian brain (Figure 1A) (1–6). Biallelic *ST3GAL5* loss-of-function variants result in systemic ganglioside deficiency, an infantile-onset neurodevelopmental syndrome characterized by intractable epileptic encephalopathy, auditory and visual impairment, global psychomotor delay, extrapyramidal movements, and untimely death. A number of pathogenic variants have been linked to the GM3 synthase deficiency syndrome (GM3SD) in populations worldwide (2, 7). Within Old Order Amish communities of North America, the incidence of GM3SD is enriched to approximately 1 per 1200 births due to a severe *ST3GAL5* c.862C > T (p.Arg288Ter) founder variant that abrogates ST3GAL5 activity and results in absence of GM3 and its most important downstream products: GM1, GD1a, GD1b, and GT1b (3).

GM3 and derivative a- and b-series gangliosides are expressed in cytosolic membranes of all mammalian cells, where they contribute to microdomain architecture and activity of intramembrane proteins (*cis* interactions), as well as ligand binding and intercellular contacts (*trans* interactions) (8). Disrupted ganglioside synthesis results in neurotoxicity from multiple overlapping mechanisms, including altered receptor interactions, abnormal cellular membrane dynamics, and reduced mitochondrial membrane potential and oxygen consumption (4, 9). Oral ganglioside replacement therapy via a powdered buttermilk supplement (G500; Auckland, New Zealand) may transiently improve growth and development during infancy, but low enteral absorption of gangliosides and their restricted transit across the blood-brain barrier (BBB) may ultimately limit the utility of dietary therapy, leading to treatment failures and loss of long-term efficacy (10). At present, no other effective treatment is available for GM3SD.

The development of novel and robust therapeutic modalities requires testing in proper animal models that genetically and phenotypically recapitulate human GM3SD. Homozygous *St3gal5*^–/–^ mice exhibit a- and b-series ganglioside deficiency, insulin hypersensitivity, and hearing loss, but in contrast to human patients, do not suffer from early mortality or clinically relevant neurological disease (11, 12). In mice, biochemical defects caused by ST3GAL5 deficiency seem to be compensated by remaining minor gangliosides species, resulting in minimal physiological alternations (Figure 1A). However, mice with KO of both *St3gal5* and *B4galnt1* are unable to synthesize any gangliosides and more closely mirror clinical hallmarks such as CNS pathology, developmental delay, motor impairment, and early death (Supplemental Figure 1; supplemental material available online with this article; https://doi.org/10.1172/jci.insight.168688DS1) (13). Thus, *St3gal5* single KO and *St3gal5/B4galnt1–*double KO mice serve as complementary models for evaluating the actions of novel therapies (Table 1).

Because GM3SD is a monogenic loss-of-function disease, gene replacement therapy may be a promising approach. Recombinant adeno-associated viruses (rAAVs) have emerged as powerful gene delivery tools for the treatment of monogenic diseases and, to date, have been tested in 17 clinical trials targeting CNS disorders (14, 15). An ideal rAAV vector should deliver its therapeutic cargo into specific target cells to restore an appropriate spatial, quantitative, and temporal pattern of protein expression. However, a limiting factor for successful and safe rAAV-mediated gene therapy is the broad tropism of common AAV serotypes. The naturally isolated AAV serotype 9 (AAV9) can cross the BBB and transduce neural tissues, but it also efficiently transduces multiple peripheral tissues such as liver, skeletal muscle, and heart (16, 17). Hepatotoxicity after high-dose systemic AAV9 delivery (18, 19), including several patient deaths due to acute liver failure (20, 21), has raised legitimate concerns about the overall safety of gene therapy. Therefore, regulating the tissue specificity of transgene delivery and expression may preserve the therapeutic benefits of rAAV while minimizing associated risks. Currently, this can be approached through a combination of variables, including route of administration (e.g., regional tissue injection), use of CNS-favorable viral capsids, and inclusion of cell type–specific promoters and tissue detargeting miRNA binding sites within the therapeutic genome sequence.

In this study, we first examined *ST3GAL5* replacement cassettes for their ability to reconstitute gangliosides in cortical neurons produced from GM3SD patient-derived induced pluripotent stem cells (iPSCs). We then administered the AAV9 vectors by intracerebroventricular (ICV) injection to *St3gal5*^–/–^ and *St3gal5*^–/–^*/B4galnt1*^–/–^ mice. Treatment with rAAV9-*ST3GAL5* extended survival, restored CNS ganglioside production, improved growth, and partially rescued motor function of experimental animals. When delivered systemically, however, this therapy led to hepatic injury and death caused by high off-target ST3GAL5 expression in the liver. We therefore designed a second-generation rAAV9 vector using a CNS-specific promoter (human Synapsin1 [Syn1]) in combination with liver-specific miRNA targeting sequences (miR-122) to optimize both transcriptional and post-transcriptional regulation (22, 23). In GM3SD mouse models, this strategy eliminated liver toxicity while preserving neurotherapeutic effects.

Finally, we examined if data from the *St3gal5*^–/–^*/B4galnt1*^–/–^–double KO mouse underrepresented the therapeutic potential of GM3SD gene therapy, because it might apply to humans and, therefore, we co-injected *St3gal5*^–/–^*/B4galnt1*^–/–^ mice with both *ST3GAL5* and *B4GALNT1* rAAV vectors. Vector co-injection completely eliminated behavior impairments in *St3gal5*^–/–^*/B4galnt1*^–/–^ mice. Overall, our study supports potentially translating safe and effective *ST3GAL5* gene therapy for clinical development.

## Results

### ST3GAL5 transgene design and in vitro expression.

*ST3GAL5-1a-2* (NM_003896) is the most abundant mRNA among 4 *ST3GAL5* mRNA variants in the human brain (Figure 1B) (24–26); we thus focused on this variant for further vector development. The first AUG start codon in *ST3GAL5-1a-*2 is in a weak translation initiation context (AUUAGUAUGC). Most ribosomes, therefore, skip the first AUG and recognize either of 2 downstream AUG sequences as the start codon (27, 28). As a result, 3 ST3GAL5 protein isoforms differing in their N-termini are produced (Figure 1B). Because of the lack of knowledge about their physiological roles, we designed and tested different human *ST3GAL5* replacement constructs carrying each ORF (Figure 1B). The codon-optimized transgenes were cloned into a ubiquitous expression cassette driven by chicken β-actin (CB) promoter with an intron (Figure 1C) and transfected into HeLa cells to confirm protein expression. We found that expression of the shortest construct (ORF3) was weak, and adding Kozak sequence GCCACC (construct KORF3) greatly enhanced expression (Figure 1D).

**Figure 2:**
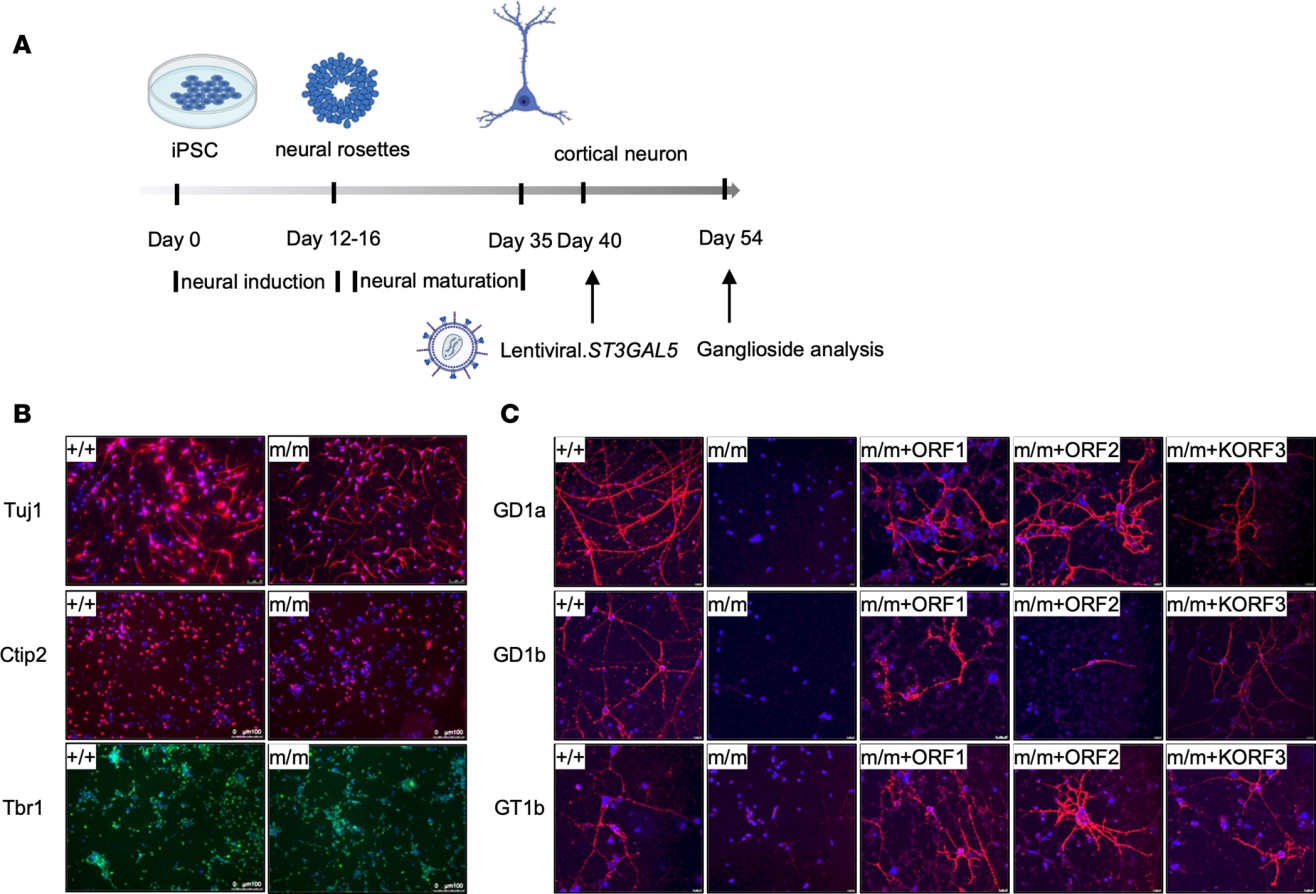
*ST3GAL5* replacement restores gangliosides production in iPSC-derived cortical neurons. (**A**) Workflow to examine restoration of ganglioside production in patients’ iPSC differentiated cortical neurons by lentiviral vectors expressing *ST3GAL5* ORFs. (**B**) Representative images of neuronal markers in *ST3GAL5^+/+^* and *ST3GAL5^mut/mut^* iPSC-differentiated cortical neurons. Neuron-specific class III β-tubulin (Tuj1) and COUP-TF-interacting protein 2 (Ctip2) are indicated by red; T-box brain transcription factor 1 (TBR1) is indicated by green, with nuclei counterstained in blue. (**C**) Representative images of major brain gangliosides in cortical neurons by lentiviral vectors expressing *ST3GAL5* ORFs. GD1a, GD1b, and GT1b are indicated by red; nuclei are counterstained in blue. *+/+*, WT; *m/m*, *ST3GAL5^mut/mut^*. Scale bars: 100 µm (**B**), 10 µm (**C**).

We next evaluated whether these *ST3GAL5* constructs could function in ganglioside synthesis in cultured neurons. To this end, we differentiated normal (*ST3GAL5^+/+^*) and patient (*ST3GAL5^E332K/E332K^*) iPSCs into cortical neurons (Figure 2, A and B) and infected them with lentiviral vectors expressing different *ST3GAL5* isoforms. Although major brain gangliosides (GD1a, GD1b, and GT1b) were absent in untreated patient neurons, they were restored after transduction of any *ST3GAL5* isoform (Figure 2C) (29). We focused on the KORF3 transgene design in future development, because M3-ST3GAL5 is the most stable isoform (24) and its small gene size (1095 base pairs) is amenable to the self-complementary AAV vector design that can further enhance vector potency when packaging capacity is limited (<2.5 kb).

### ICV injection of rAAV9-ST3GAL5.v1 improved biochemical and phenotypic abnormalities in GM3SD mouse models.

**Figure 3:**
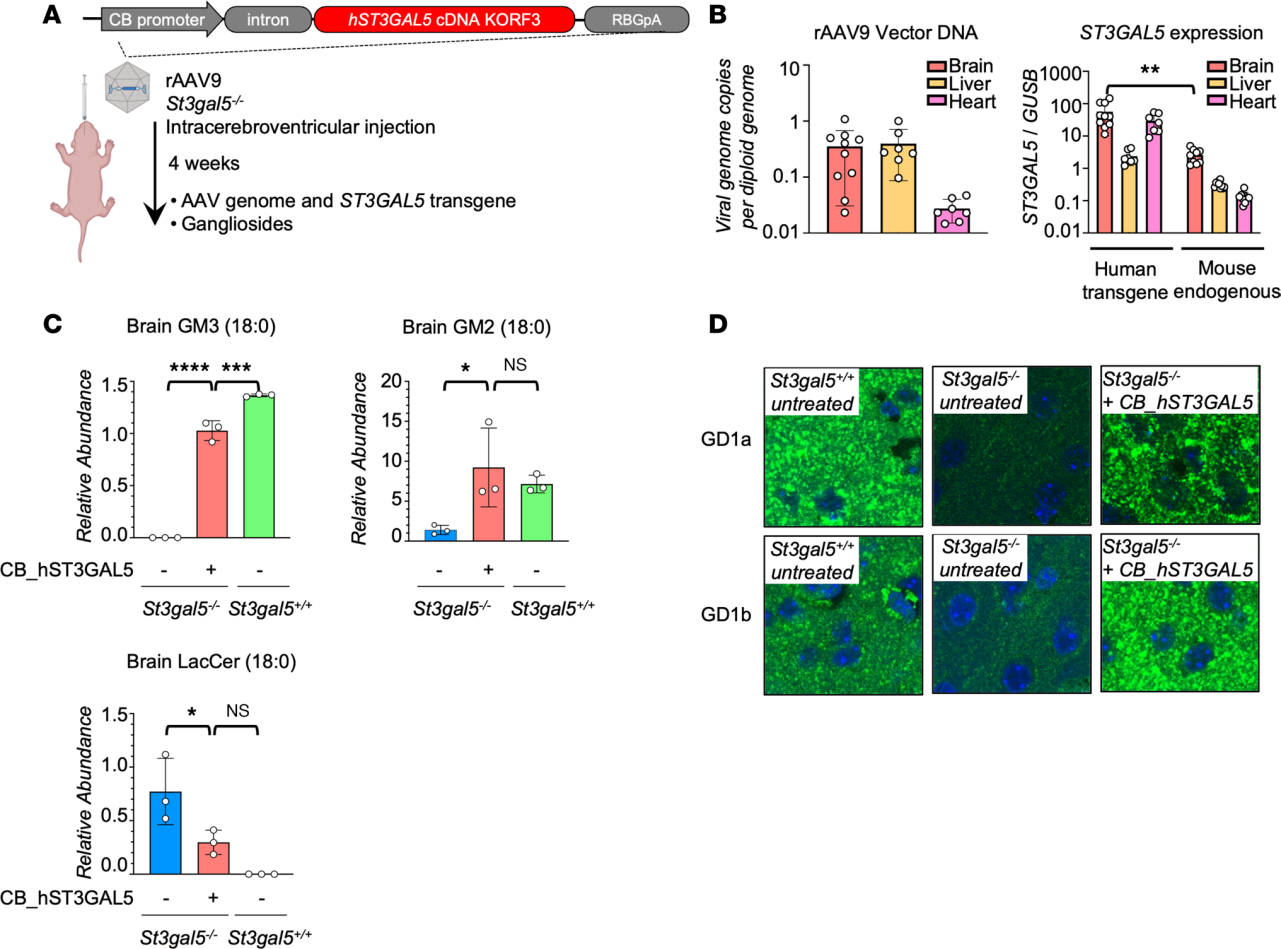
ICV delivery of *ST3GAL5* restores gangliosides production in *St3gal5^–/–^* mouse model. (**A**) Schematic of ICV delivery of ubiquitous human *ST3GAL5* cDNA Kozak ORF3 (KORF3) in *St3gal5*^–/–^ mouse model. (**B**) ddPCR quantification of rAAV9 vector genome and human *ST3GAL5* transgene in the brain, liver, and heart of rAAV9.CB.hST3GAL5-treated *St3gal5*^–/–^ mice. Mouse endogenous *St3gal5* mRNA was quantified from brain, liver, and heart of *St3gal5^+/+^* mice. Data are reported as the mean ± SD of 7–10 animals/group. Statistical analysis was performed by 2-tailed *t* test. (**C**) Mass spectrometry quantification of GM3 (18:0), GM2 (18:0), and LacCer (18:0) from the brain of *St3gal5^+/+^* and *St3gal5*^–/–^ mice, with (+) or without (–) rAAV9.CB.hST3GAL5 treatment. Data are reported as the mean ± SD of 3 animals/group. Statistical analysis was performed by 1-way ANOVA, followed by Sidak’s multiple comparisons test. (**D**) Representative images of major brain gangliosides in cortex of *St3gal5^+/+^* and *St3gal5*^–/–^ mice, with (+) or without (–) rAAV9.CB.hST3GAL5 treatment. GD1a and GD1b are indicated by green; nuclei are counterstained in blue. Magnification, 63***×***. Scale bar: 3 µm. Quantification is shown in Supplemental Figure 2. *P < 0.05, **P < 0.01, ***P < 0.001, ****P < 0.0001.

Encouraged by in vitro results, we generated an AAV9 vector carrying a CB promoter-driven *ST3GAL5* construct (rAAV9-ST3GAL5.v1) to assess therapeutic efficacy in mice after in vivo delivery. We first treated *St3gal5*^–/–^ mice by unilateral ICV injection of 3 × 10^10^ genome copies (gcs) per pup at P1, and we euthanized animals 4 weeks after injection (Figure 3A). The *ST3GAL5* transgene was detected in brain, liver, and heart, where it induced tissue mRNA expression in excess of endogenous levels (Figure 3B). GM3 and its derivatives (GM2, GD1a, and GD1b) were undetectable in the *St3gal5*^–/–^ brain. ICV injection of rAAV9-ST3GAL5.v1 restored these a- and b-series gangliosides to WT levels, concomitant with substantial clearance of lactosylceramide (LacCer), the proximate substrate for GM3 synthase (Figure 3, C and D, and Supplemental Figure 2). However, ganglioside deficiency persisted in serum, likely because vector genomes delivered by ICV injection did not sufficiently penetrate peripheral tissues (Supplemental Figure 3A). Note that total GM1 (consisting of GM1a and GM1b) was not affected by the *St3gal5* KO (Supplemental Figure 3B). Because gangliosides are 10- to 30-fold more abundant in human brain than in any other tissue (30), we postulated that ganglioside restoration in CNS was the key to preventing neurological morbidity in GM3SD animals.

*St3gal5*^–/–^ mice have hearing loss but do not exhibit many of the other neurological deficits characteristic of GM3SD in humans. For testing vector effectiveness, we therefore used the *St3gal5*^–/–^*/B4galnt1*^–/–^ mouse model, which models key aspects of the human GM3SD phenotype, including reduced survival, growth failure, motor impairments, and neuropathology. These animals were benchmarked to *St3gal^+/–^/B4galnt1*^–/–^ mice, which were from the same litter as *St3gal5*^–/–^*/B4galnt1*^–/–^ and did not exhibit significant neurological morbidity during the period of experimental observation (Supplemental Figure 4A). ICV administration of rAAV9-ST3GAL5.v1 to newborn *St3gal5*^–/–^*/B4galnt1*^–/–^ pups (3 × 10^10^ gcs; P1) extended their survival up to 300 days (median survival: untreated, 18 days; treated, 56 days) (Supplemental Figure 4B), improved growth, and partially restored motor function as assessed by the negative geotaxis test (Supplemental Figure 4, C–F). Taken together, these results demonstrated that neonatal ICV injection of rAAV9-ST3GAL5.v1 was well tolerated and could restore endogenous cerebral ganglioside production but did not fully alleviate GM3SD disease in *St3gal5*^–/–^*/B4galnt1*^–/–^ mice.

### Systemic delivery of rAAV9-ST3GAL5.v1 caused liver toxicity.

although ICV injection considerably reduced disease burden in murine models, we wondered if systemic delivery could have further advantages. Specifically, systemic vector delivery has the potential to more broadly and evenly distribute AAV9 vector throughout the neuraxis, taking advantage of a naturally dense capillary network that perfuses the mammalian CNS (31), and could deliver therapeutic genomes to peripheral neural tissues (e.g., peripheral nerve axons and Schwann cells) that express, and may be functionally dependent upon, complex gangliosides (32–34). Accordingly, we administered rAAV9-ST3GAL5.v1 to P1 *St3gal5*^–/–^ pups by facial vein injection using 3 × 10^11^ gcs/pup, and treated *St3gal5*^+/+^ littermates in parallel as controls.

**Figure 4:**
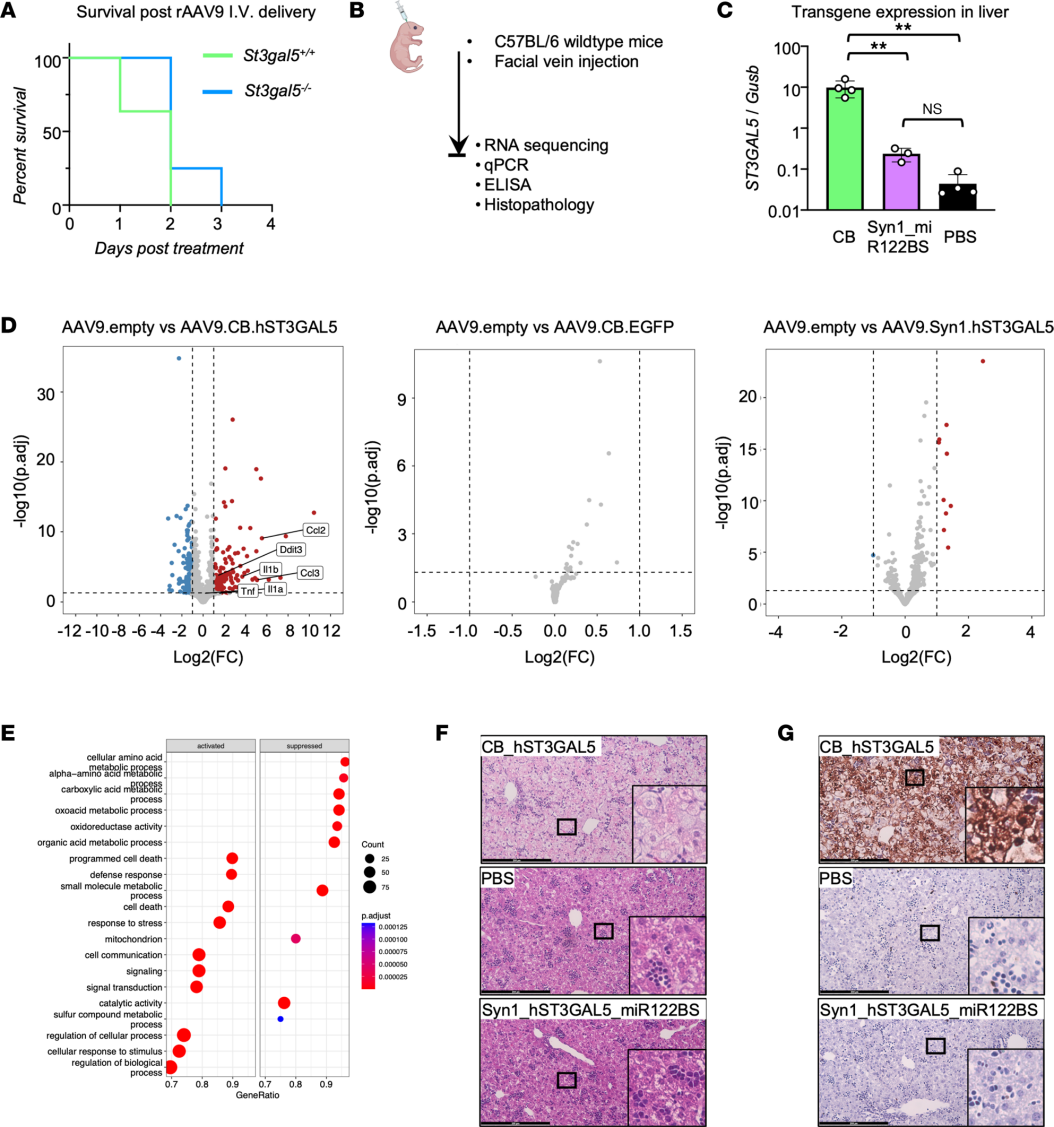
Liver detargeting eliminates ST3GAL5 overexpression–induced toxicity. (**A**) Median survival after AAV9.CB.hST3GAL5 i.v. delivery. Data are plotted as probability of survival from 4 to 11 animals. (**B**) Schematic of facial-vein delivery of AAV9.CB.hST3GAL5, or AAV9. EGFP, or AAV9.empty, or AAV9.Syn1.hST3GAL5.miR122BS, or PBS in WT mice. (**C**) ddPCR quantification of human ST3GAL5 cDNA in the liver of WT mice with rAAV9.CB.hST3GAL5 or rAAV9.hSyn1.hST3GAL5.miR122BS treatments and endogenous mouse St3gal5 from PBS treatment. Data are reported as the mean ± SD of 3–4 animals/group. Statistical analysis was performed by 1-way ANOVA with post hoc Tukey multiple comparison test. (**D**) Volcano plots showing differentially expressed genes in mouse livers. Blue indicates reduced expression; red indicates increased expression; grey indicates ns difference. Adjusted *P* ≤ 0.05; fold change ≥ 2. (**E**) Graph depicting significantly enriched pathways for differentially expressed genes between livers from WT mice injected with PBS and rAAV9.CB.hST3GAL5 using gene set enrichment analysis. (**F**) Representative images of H&E staining of liver sections from WT mice injected with rAAV9.CB.hST3GAL5 or PBS or rAAV9.hSyn1.hST3GAL5.miR122BS. (**G**) Representative images of TUNEL staining of liver sections from WT mice injected with rAAV9.CB.hST3GAL5 or PBS or rAAV9.hSyn1.hST3GAL5.miR122BS. *P < 0.05, **P < 0.01. p.adjust, adjusted *P* value. Scale bar: 200 µm.

All treated *St3gal5*^–/–^ and *St3gal5^+/+^* mice unexpectedly died within 3 days after injection (Figure 4A). To understand why, we injected WT pups with therapeutic vector, capsids containing cDNA for EGFP, empty capsids (AAV9.empty), or PBS (Figure 4B). We found that in WT mice systemically treated with rAAV9-ST3GAL5.v1, expression of *ST3GAL5* in liver was elevated more than 100-fold relative to mouse endogenous *St3gal5* level (Figure 4C), accompanied by activation of cellular death and defense response (Figure 4, D and E). We confirmed RNA-Seq results with quantitative PCR (qPCR) and ELISA, which revealed consistent activation of pro-inflammatory cytokines (TNF-α, IL-1α, CCL2, and CCL3) (Supplemental Figure 5, A and B). These molecular changes were accompanied by cellular liver pathology, including hepatocyte swelling (Figure 4F) and cell death (Figure 4G). Moreover, the toxicity was also seen in liver of *St3gal5*^–/–^ mice receiving rAAV9-ST3GAL5.v1 (Supplemental Figure 6, A–D) but not in other peripheral organs (Supplemental Figure 6, E and F). We thus speculated that overexpression of *hST3GAL5* transgene in liver and its attendant cytopathic effects played a direct role in lethal toxicity of systemically administered rAAV9-ST3GAL5.v1.

### Optimized ST3GAL5 vector construct with spatial regulation eliminated liver toxicity associated with systemic administration.

We reasoned that CNS-restricted and liver-detargeted expression of an *ST3GAL5* transgene might preserve therapeutic efficacy while eliminating hepatotoxicity. We therefore designed a spatially regulated expression cassette that included human Syn1 promoter (35, 36) to drive neuronal expression at the transcriptional level, combined with miR-122 binding sites in the 3′UTR, which silence transgene expression in hepatocytes at the post-transcriptional level (Figure 4C) (22, 23). We named this refined construct ST3GAL5.v2 and packaged it into AAV9. Following the same P1 facial vein injection paradigm in WT pups, transgene expression from rAAV9-ST3GAL5.v2 was greatly reduced in liver tissue, and all animals survived with no evidence of liver inflammation, cytopathology, or transcriptomic derangements (Figure 4 and Supplemental Figure 5).

Interestingly, we noticed that packaging the first-generation construct (rAAV9-ST3GAL5.v1) consistently resulted in low titers (1 × 10^12^ to 4 × 10^12^ gcs/mL), likely due to transgene toxicity in HEK293 cells during the manufacturing process. In contrast, rAAV9-ST3GAL5.v2 was routinely produced at higher titers of 0.8 × 10^13^ to 1.5 × 10^13^ gcs/mL (Supplemental Figure 7). We therefore concluded that by tuning tissue specificity, the optimized second-generation construct design eliminated both hepatotoxicity and the manufacturing bottleneck, serving as a clinically translatable candidate for studies that followed.

### ICV injection of rAAV9-ST3GAL5.v2 improved biochemical and phenotypic abnormalities in GM3SD mouse models.

**Figure 5:**
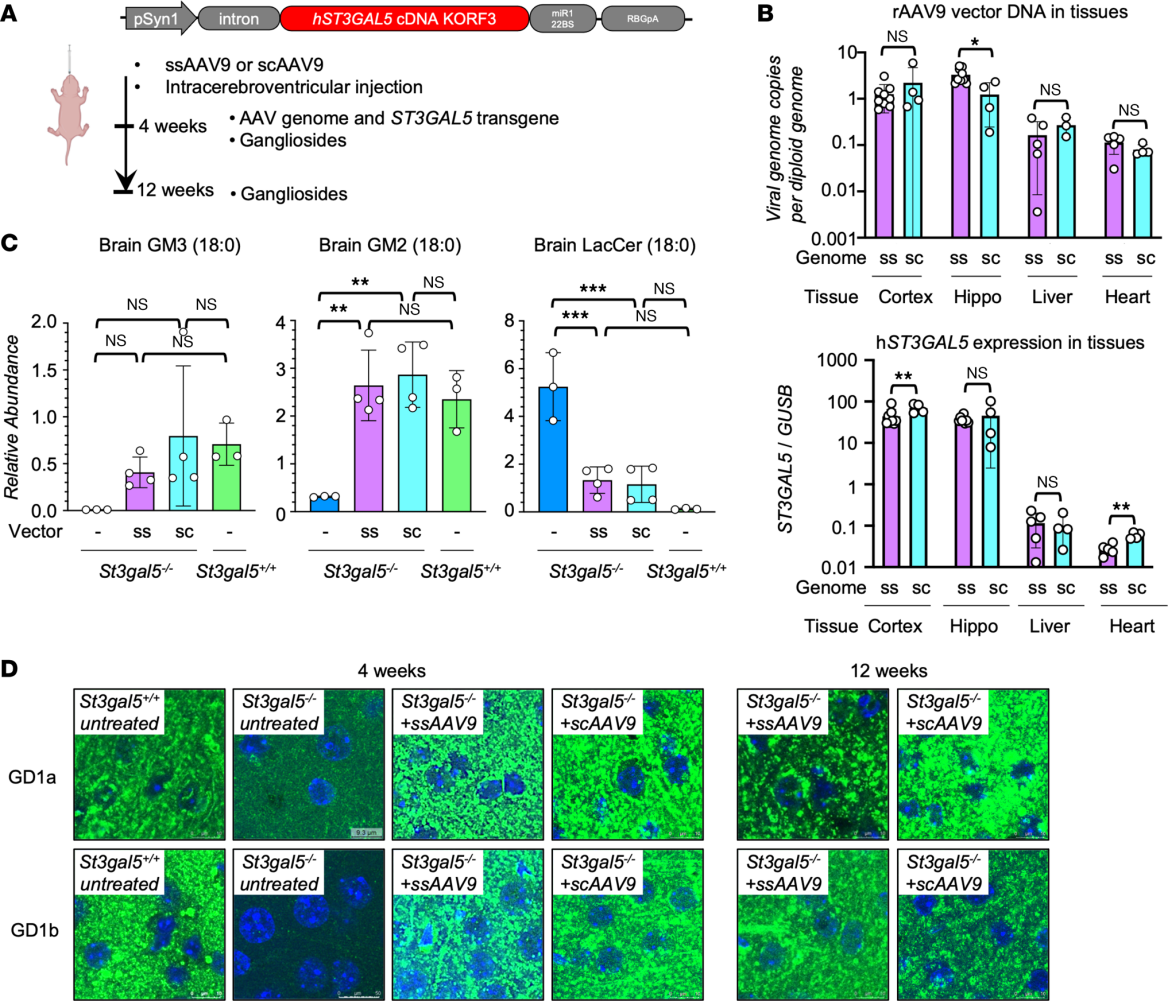
Second-generation of *ST3GAL5* replacement vector restores ganglioside production in *St3gal5^–/–^* mouse model. (**A**) Schematic of ICV delivery of neuron-specific human *ST3GAL5* KORF3 (ST3GAL5.v2) in the *St3gal5*^–/–^ mouse model. (**B**) ddPCR quantification of rAAV9 genome and human *ST3GAL5* cDNA in the cortex, hippocampus (Hippo), liver, and heart of ssAAV9.ST3GAL5.v2- or scAAV9.ST3GAL5.v2-treated *St3gal5*^–/–^ mice. Data are reported as the mean ± SD of 4–9 animals/group. Statistical analysis was performed by 2-tailed Student’s *t* test. (**C**) Mass spectrometry quantification of GM3 (18:0), GM2 (18:0), and LacCer (18:0) from the brain of *St3gal5^+/+^* and *St3gal5*^–/–^ mice, with ssAAV9.ST3GAL5.v2 or scAAV9.ST3GAL5.v2 or no treatment. Data are reported as mean ± SD of 3–4 animals/group. Statistical analysis was performed by 1-way ANOVA, followed by Sidak’s multiple comparisons test. (**D**) Representative images of major brain gangliosides in cortex of *St3gal5^+/+^* and *St3gal5*^–/–^ mice, with ssAAV9.ST3GAL5.v2 or scAAV9.ST3GAL5.v2 or no treatment. GD1a and GD1b are indicated by green; nuclei are counterstained in blue. Quantification is shown in Supplemental Figure 2. *P < 0.05, **P < 0.01, ***P < 0.001. sc, self-complementary; ss, single-stranded. Scale bar: 10 µm.

We cloned the ST3GAL5.v2 construct in self-complementary (sc) configuration to facilitate faster and stronger expression as compared with the single-stranded (ss) transgene (Figure 5A and Supplemental Figure 8) (37, 38). After P1 ICV injection in *St3gal5*^–/–^ mice, scAAV9- and ssAAV9-ST3GAL5.v2 led to comparable levels of transgene expression in hippocampus 4 weeks after injection, whereas scAAV9 slightly outperformed ssAAV9 in the cerebral cortex (Figure 5B). Both vectors normalized the brain ganglioside profile in *St3gal5*^–/–^ mice up to 12 weeks after injection (Figure 5, C and D), although neither corrected circulating gangliosides, consistent with the neuron-specific expression design (Supplemental Figure 9).

**Figure 6:**
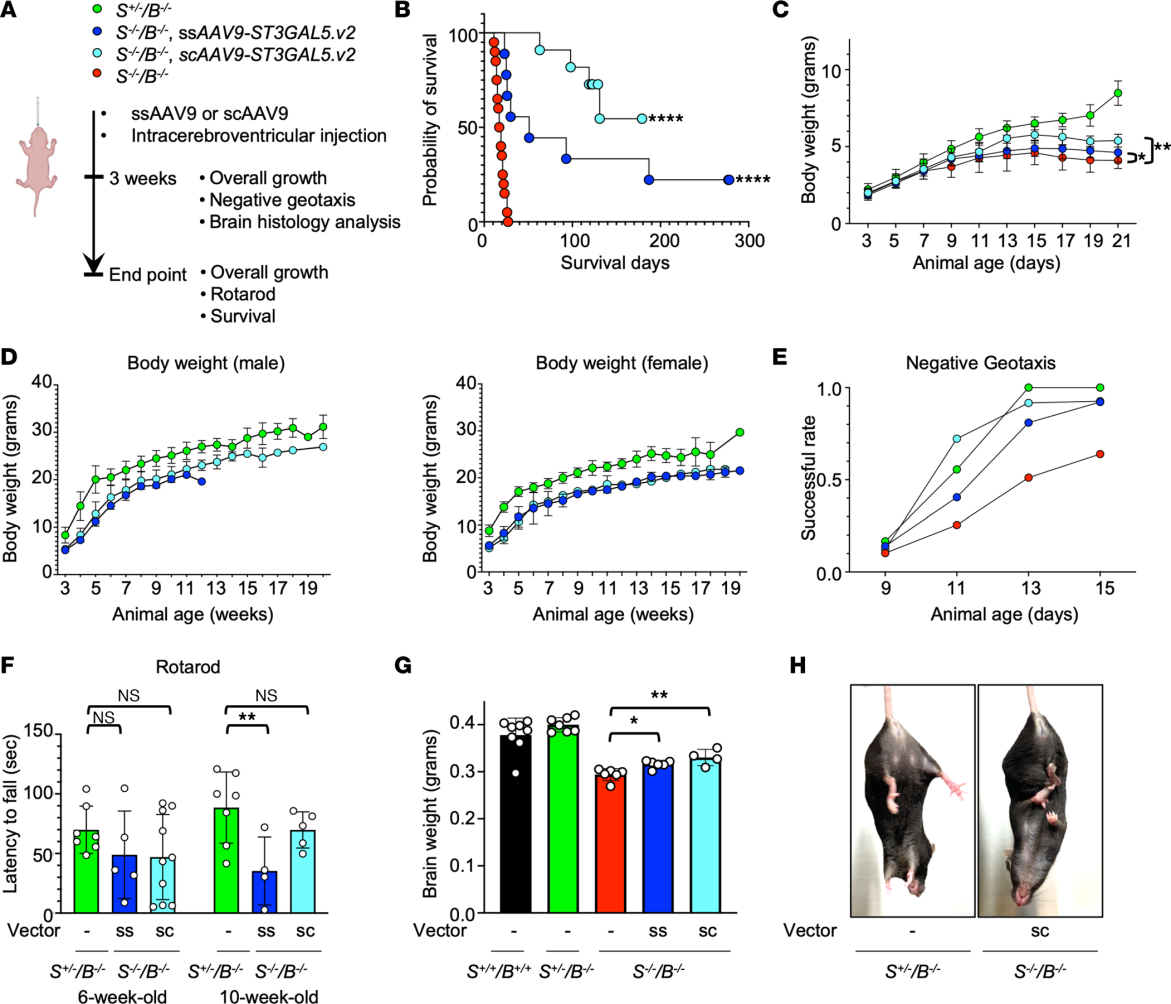
Second-generation of *ST3GAL5* replacement vector rescues phenotypical changes in *St3gal5^–/–^/B4galnt1^–/–^* mouse model. (**A**) Schematic of ICV delivery of rAAV-ST3GAL5.v2 in the *St3gal5*^–/–^*/B4galnt1*^–/–^ mouse model. (**B**) Median survival of *St3gal5*^–/–^*/B4galnt1*^–/–^ mice with ssAAV9.ST3GAL5.v2 or scAAV9.ST3GAL5.v2 or no treatments. Data from 7 to 20 animals are plotted as the probability of survival. Statistical analysis was performed by log-rank (Mantel-Cox) test. (**C**) A time-course BW of postnatal pups aged 3–21 days old. Data are reported as the mean ± SD of 10 animals. Statistical analysis was performed by 2-way ANOVA, followed by Sidak’s multiple comparisons test. (**D**) BW at postweaning stage. Data are reported as the mean ± SD of 5–8 animals. (**E**) Negative-geotaxis success rate of postnatal pups aged 9–15 days old. Data are normalized from 10 animals. (**F**) Quantification of rotarod assay for *St3gal5*^+/–^*/B4galnt1*^–/–^ mice and ssAAV9.ST3GAL5.v2 or scAAV9. T3GAL5.v2 treated *St3gal5*^–/–^*/B4galnt1*^–/–^ mice at 6 or 10 weeks old. Data are reported as the mean ± SD of 4–7 animals. Statistical analysis was performed by 1-way ANOVA, followed by Sidak’s multiple comparisons test. (**G**) Quantification of brain weight from WT mice or *St3gal5^+/–^/B4galnt1*^–/–^ mice or *St3gal5*^–/–^*/B4galnt1*^–/–^ mice with ssAAV9.ST3GAL5.v2 or scAAV9.ST3GAL5.v2 or no treatments at 3 weeks old. Data are reported as the mean ± SD of 4–8 animals. Statistical analysis was performed by 1-way ANOVA, followed by Sidak’s multiple comparisons test. (**H**) Representative images of mouse hindlimb clasping from in an *St3gal5*^–/–^*/B4galnt1*^–/–^ mouse with scAAV9.ST3GAL5.v2 treatment or an *St3gal5^+/–^/B4galnt1*^–/–^ mouse. *P < 0.05, **P < 0.01, ****P < 0.0001.

**Figure 7:**
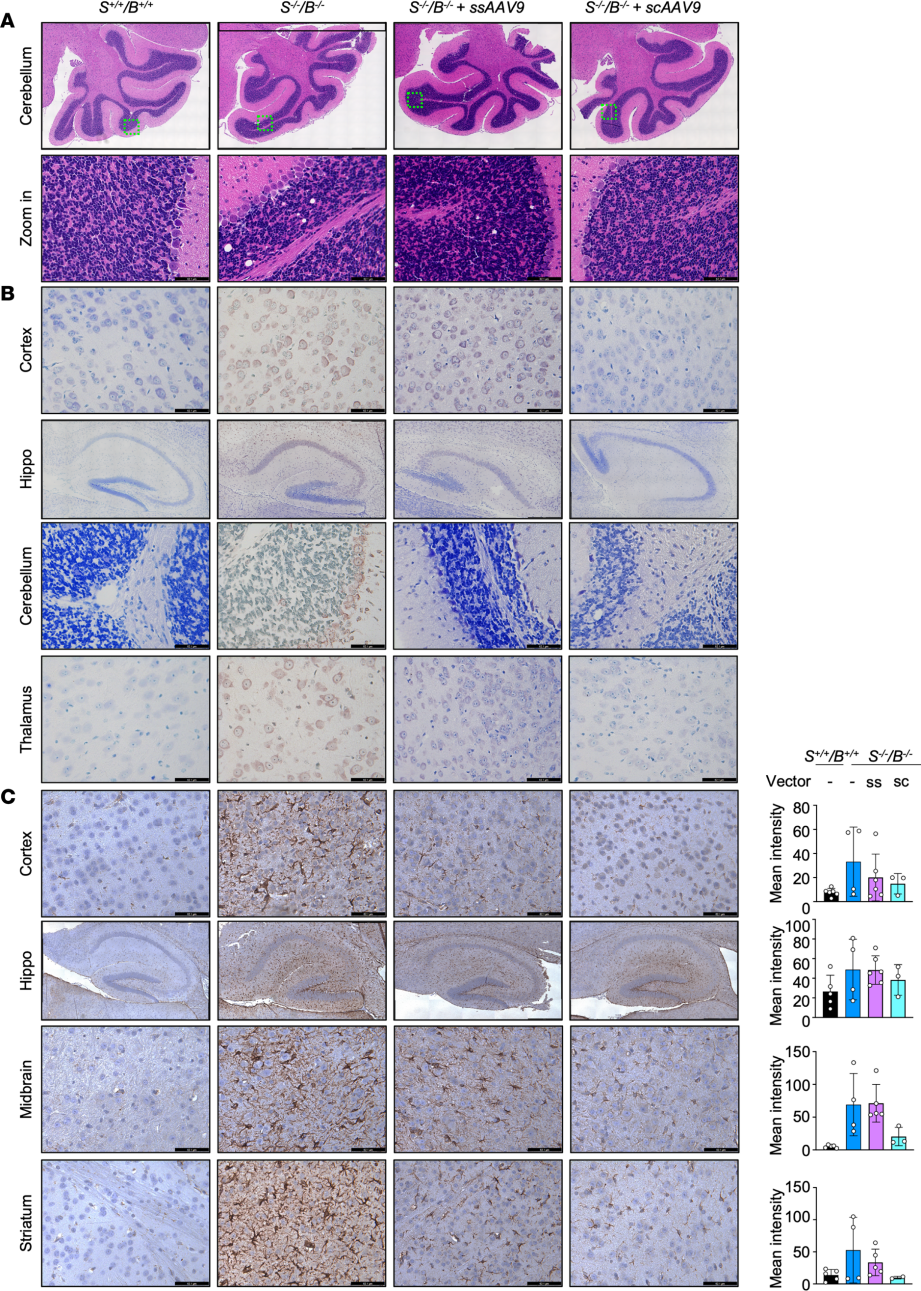
Second-generation of *ST3GAL5* replacement vector rescues brain histology in the *St3gal5^–/–^/B4galnt1^–/–^* mouse model. (**A**) Representative images of H&E staining of cerebellum sections from WT mice and *St3gal5*^–/–^*/B4galnt1*^–/–^ mice with ssAAV9.ST3GAL5.v2 or scAAV9. ST3GAL5.v2 or no treatments. Black rectangle, zoom-in area. (**B**) Representative images of TUNEL staining of brain sections (cortex, hippocampus [hippo], cerebellum) from WT and *St3gal5*^–/–^*/B4galnt1*^–/–^ mice with ssAAV9.ST3GAL5.v2 or scAAV9.ST3GAL5.v2 or no treatments. (**C**) Representative images and quantification of anti-GFAP immunostaining of brain sections (cortex, hippocampus, midbrain) from WT and *St3gal5*^–/–^*/B4galnt1*^–/–^ mice with ssAAV9.ST3GAL5.v2 or scAAV9.ST3GAL5.v2 or no treatments. Mean intensity was quantified by Fiji. Data are reported as the mean ± SD of 3–5 animals. Statistical analysis was performed by 1-way ANOVA, followed by Sidak’s multiple comparisons test. sc, self-complementary; ss, single-stranded. Scale bar: 62.1 µm.

Using the same P1 ICV injection paradigm, we next treated *St3gal5*^–/–^*/B4galnt1*^–/–^ pups with either scAAV9- or ssAAV9-ST3GAL5.v2 (Figure 6A). Both vectors significantly extended animal survival (median survival: untreated, 19 days; ssAAV9, 51 days; scAAV9: 101 days) (Figure 6B), partially restored body growth (Figure 6, C and D), and largely normalized motor function, as revealed by negative geotaxis and rotarod tests (Figure 6, E and F). Importantly, both treatments improved brain growth (Figure 6G) while reducing or eliminating neuropathological changes such as cerebellar vacuolization (Figure 7A), neuronal cell death (Figure 7B), and astrogliosis (Figure 7C) across multiple brain regions; scAAV9 consistently outperformed ssAAV9 in all histopathological assessments. Furthermore, scAAV9-ST3GAL5.v2 vector treatment at later age (P4) could also extend animal survival and partially restore body growth (Supplemental Figure 10), highlighting a strong translational relevance.

**Figure 8:**
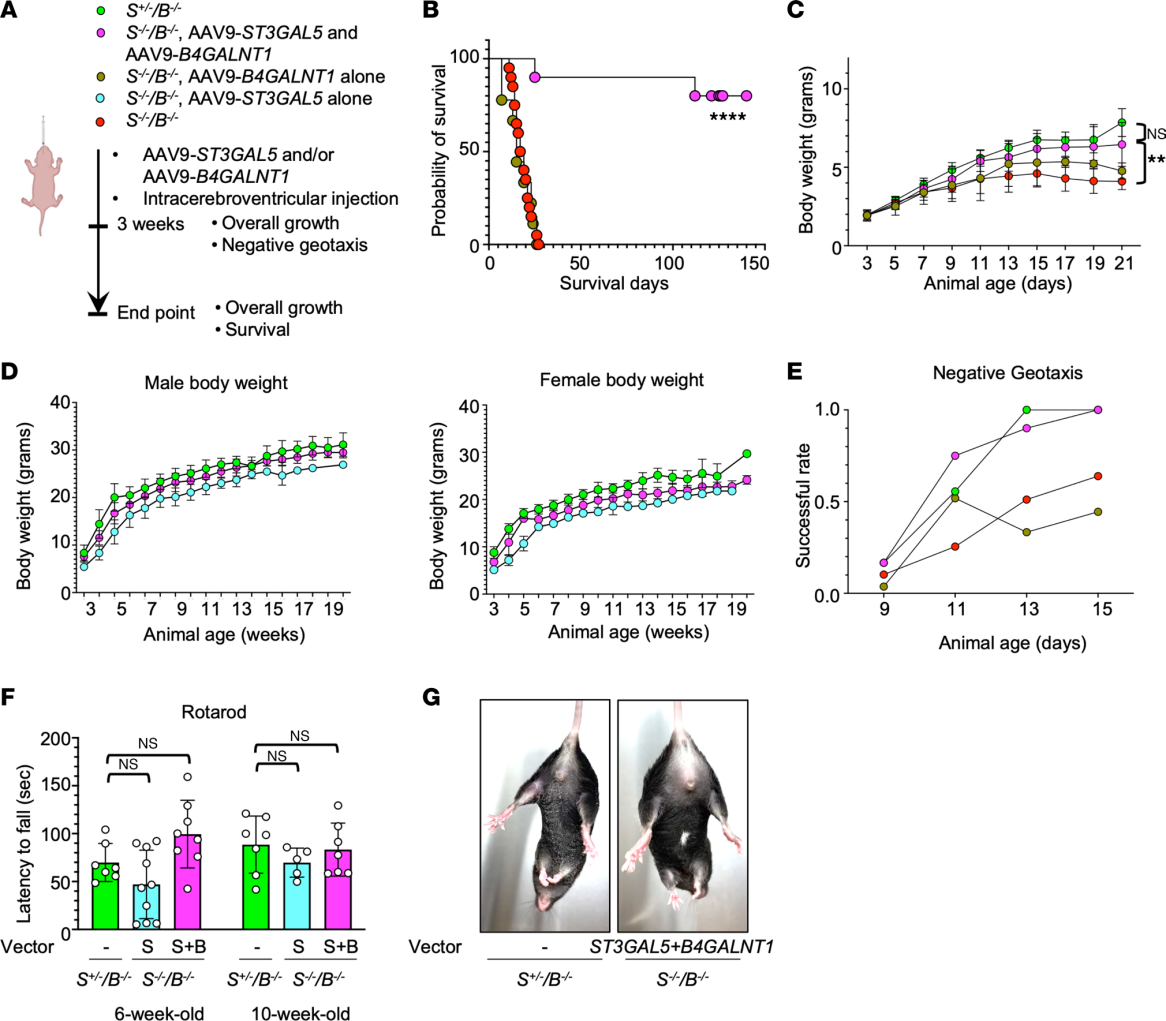
Codelivery of *ST3GAL5* and *B4GALNT1* vectors normalize the *St3gal5^–/–^/B4galnt1^–/–^* mouse model. (**A**) Schematic of ICV codelivery of AAV vectors expressing *ST3GAL5* and *B4GALNT1* cDNA, respectively, in the *St3gal5*^–/–^*/B4galnt1*^–/–^ mouse model. (**B**) Median survival of *St3gal5*^–/–^*/B4galnt1*^–/–^ mice with or without co-delivery of *ST3GAL5* and *B4GALNT1*. Data from 8 to 20 animals are plotted as probability of survival. Statistical analysis was performed by log-rank (Mantel-Cox) test. (**C**) A time-course BW of postnatal pups aged 3–21 days old. Data are reported as the mean ± SD of 10 animals. Statistical analysis was performed by 2-way ANOVA, followed by Sidak’s multiple comparisons test. (**D**) BW of male and female mice at the postweaning stage. Data are reported as the mean ± SD of 3–5 animals. (**E**) Negative-geotaxis success rate of postnatal pups aged 9–15 days old. Data are normalized from 7–9 animals. (**F**) Quantification of rotarod assay for *St3gal5^+/–^/B4galnt1*^–/–^ mice and scAAV9.ST3GAL5.v2 or dual vector–treated *St3gal5*^–/–^*/B4galnt1*^–/–^ mice at 6 and 10 weeks old. Data are reported as the mean ± SD of 5–8 animals. (**G**) Representative images of mouse hindlimb from the *St3gal5*^–/–^*/B4galnt1*^–/–^ mouse with dual-vector treatment or the *St3gal5^+/-^/B4galnt1*^–/–^ mouse. **P < 0.01, ****P < 0.0001.

Nevertheless, *St3gal5*^–/–^*/B4galnt1*^–/–^ mice that survived after scAAV9-ST3GAL5.v2 treatment continued to exhibit hindlimb clasping, a sign of motor dysfunction not seen in their *St3gal5^+/–^/B4galnt1*^–/–^ littermates (Figure 6H). This might reveal a fundamental limitation of the sequential double KO animal model; that is, isolated KO of *B4galnt1* in mice has independent neuropathological effects (39, 40) that cannot be fully rescued by replacing *ST3GAL5* alone. To test this hypothesis, we generated ssAAV9-CB-B4GALNT1 and co-delivered it with scAAV9-ST3GAL5.v2 by ICV administration. Indeed, this dual-vector treatment regimen completely rescued lethality, growth retardation, hindlimb clasping, and motor impairment in *St3gal5*^–/–^*/B4galnt1*^–/–^ mice (Figure 8). These encouraging results suggest that using the *St3gal5*^–/–^*/B4galnt1*^–/–^ mouse model to test *ST3GAL5* gene replacement may be overly stringent and underrepresent the clinical potential of scAAV9 *ST3GAL5* gene replacement vectors.

### IV injection of rAAV.PHP.eB-ST3GAL5.v2 improved phenotypic abnormalities in GM3SD mouse models.

To examine whether systemically delivered scAAV9-ST3GAL5.v2 could achieve broader brain transduction and better therapeutic efficacy without causing liver toxicity, we treated *St3gal5*^–/–^*/B4galnt1*^–/–^ pups with 3 × 10^11^ gcs on P1 by facial vein injection. Although we did not observe the acute lethality associated with systemic administration of first-generation vectors, i.v. scAAV9-ST3GAL5.v2 showed limited efficacy with regard to survival (median survival: 34 days), growth, and motor function (Supplemental Figure 11, B–D), likely due to low *ST3GAL5* expression and poor ganglioside reconstitution in the brain as compared with ICV injection (Supplemental Figure 11, E–G). We thus packaged the v2 construct into PHP.eB, an engineered AAV capsid that penetrates the murine BBB more efficiently than AAV9 (41). Under the same systemic administration regimen, ssAAV.PHP.eB-ST3GAL5.v2 led to higher transgene expression in the brain, robust CNS ganglioside restoration, and better phenotypic rescue by all measurements (Supplemental Figure 11, B–G). Taken together, these data underscore the importance of restoring ganglioside synthesis in CNS, particularly in neurons, for ameliorating the GM3SD phenotype.

## Discussion

**Figure 9:**
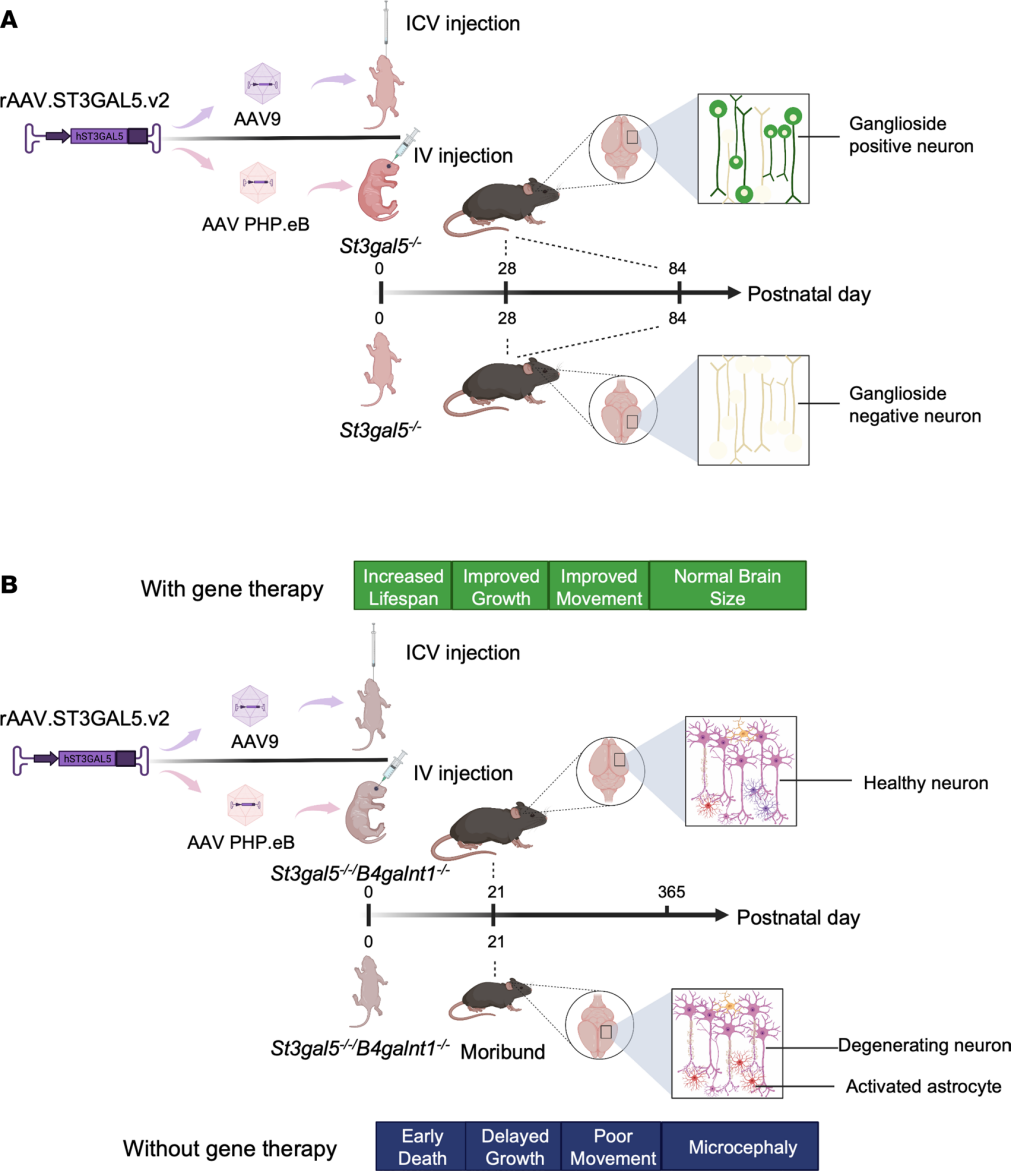
Summary of spatially regulated rAAV-mediated *ST3GAL5* delivery in GM3SD mouse models. (**A**) Schematic of ICV delivery of AAV9 vectors or i.v. delivery of PHP.eB vectors expressing *ST3GAL5* in an *St3gal5*^–/–^ mouse model. (**B**) Schematic of ICV delivery of AAV9 vectors or i.v. delivery of PHP.eB vectors expressing *ST3GAL5* in an *St3gal5*^–/–^*/B4galnt1*^–/–^ mouse model.

In this proof-of-concept study, we show that rAAV-mediated *ST3GAL5* gene replacement restores cerebral ganglioside synthesis, ameliorates neuropathology, and improves motor function in 2 different murine models of human GM3SD (*St3gal5*^–/–^ and *St3gal5*^–/–^*/B4galnt1*^–/–^*)*. Of note, both ICV and i.v. routes of administration provided meaningful benefits in animal models, illustrating that CNS-directed *ST3GAL5* replacement holds promise for further clinical development (Figure 9).

Developmental and functional differences in ganglioside biology of mice, as compared with humans, present a significant experimental challenge. Humans with severe, biallelic loss-of-function mutations in *ST3GAL5* exhibit complete absence of GM3 and its downstream derivatives in plasma and, presumably, brain tissue, and present with epileptic encephalopathy and psychomotor stagnation within a few months of life. A similar enzyme disruption in *St3gal5*^–/–^ mice leads to tissue deficiency of GM3 but a comparatively mild pathological and behavioral phenotype. Although the major documented phenotype in these mice is hearing loss, we were unable to investigate this further due to limitations in our techniques and expertise. In the absence of GM3 synthase, LacCer is shunted into alternative biosynthetic pathways for the production of O-series gangliosides (Figure 1) from human patients and *St3gal5*^–/–^ mice (11, 42). It is unclear yet if the complex gangliosides are redundant for maintaining membrane physics and signal transduction in different species. Further thorough analysis of gangliosides and related glycosphingolipids may help understand this discrepancy. On the other hand, a more phenotypically relevant murine model requires simultaneous disruption of 2 serial enzymes in the ganglioside synthetic pathway, *St3gal5* and *B4galnt1*. These double KO mice exhibit severe neuropathology and functional deficits concordant with human GM3SD, but suffer from abiding and functionally relevant *B4GALNT1* deficiency after successful *ST3GAL5* replacement. Thus, although *St3gal5*^–/–^*/B4galnt1*^–/–^ mice allow us to test the efficacy of different *ST3GAL5* replacement vectors, they may underestimate the therapeutic potential of such vectors for treatment of human GM3SD. This scenario underscores the importance of using relevant animal models in preclinical gene therapy studies. Modeling GM3SD in larger gyrencephalic species, such as pigs or sheep, might prove more informative for future studies (43).

A number of other technical hurdles exist for the treatment of neurological diseases via gene replacement. For example, it is increasingly clear that for many neurogenetic deficiencies, successful treatment will depend on efficient and even delivery of transgene across the neuraxis coupled to a pattern of expression that approximates the natural distribution, abundance, and developmental timing of WT protein. As an example, our first-generation vector induced an active unfolded protein response and severe hepatotoxicity caused by off-target hepatic overexpression of ST3GAL5. Organ toxicity caused by transgene overexpression has been observed in other preclinical disease models. For example, adult mice administered AAV9-*MECP2* replacement vectors at 10^12^ gcs/mouse develop liver damage with drastically elevated liver transaminases levels and disorganized liver architecture. Similar to our findings reported here, the damage is associated with overabundant expression of MECP2 and apoptosis triggered by UPR (44). In another murine system, neonatal mice receiving ICV delivery of AAV9-SMN at 5 × 10^10^ gcs/g develop late-onset, dose-dependent motor dysfunction, impaired proprioception, and neurodegenerative changes due to overexpression of SMN and subsequent RNA dysregulation (45).

Working toward safer and more efficacious therapy, we combined a neuron-specific human Syn1 promoter with a liver-specific miR122 targeting sequence in our second-generation vector. This design prevented ST3GAL5 overexpression in liver and thereby eliminated liver toxicity. The same principle of using facilitative cell-specific promoters coupled to inhibitory miRNA binding sites could be applied more broadly for achieving refined expression specificity (46–48). In general, we believe that optimizing spatial and temporal regulation of transgene expression will enable safer and more effective systemic gene therapy for a number of neurogenetic disorders in humans, and GM3SD provides an important experimental model to test this idea. Furthermore, scAAV, which uses a mutated inverted terminal repeat (ITRs) to generate an intramolecular, double-stranded genome configuration, allows for faster and higher gene expression but limits the packaging capacity to half that of the ssAAV genome (37, 38, 49). Indeed, the stronger ST3GAL5 expression driven by scAAV restored ganglioside production and improved phenotypical abnormality more efficiently in the present study.

Last, to identify the most effective and clinically translatable route for administering *ST3GAL5* to CNS cells, we tested both ICV and i.v. routes commonly used in current clinical trials (50). ICV injection bypasses the BBB, similar in principle to the more spatially delimited intrathecal injection of nusinersen (51), but does not leverage the dense cytological distribution of natural CNS capillaries. We found that ICV delivery of *ST3GAL5* at a clinically feasible dose (2 × 10^13^gcs/kg) achieved promising therapeutic outcomes in neonatal mice. In contrast, i.v.-injected vector at a 10-fold higher dose (2 × 10^14^gcs/kg) did not restore ganglioside production or prevent disease manifestations. Thus, among these preclinical dosing paradigms, ICV injection appeared superior at a clinically feasible dose.

Highly neurotropic AAV capsids, delivered systemically via CNS capillaries, may be key to achieving much broader CNS distribution. As a proof-of-concept, we tested i.v. delivery of *ST3GAL5* encapsulated in PHP.eB, an engineered capsid that crosses the murine BBB more efficiently than AAV9. The biochemical and phenotypic results were promising. Systemic delivery of PHP.eB is not clinically translatable because of its species- and strain-specific characteristics, but our results can inform future studies of CNS favorable capsids.

In conclusion, AAV-mediated CNS gene transfer with *ST3GAL5* at a clinically relevant dose provides significant biochemical and therapeutic benefits with limited off-target toxicity. Notwithstanding limitations of current murine models, our second-generation scAAV9-ST3GAL5.v2 replacement vector is a promising candidate for further clinical development of *ST3GAL5* gene therapy.

## Methods

### Study design

The primary goal of this study was to develop an rAAV-mediated *ST3GAL5* replacement therapy to treat GM3SD. Our experimental approach combined cells derived from patients with GM3SD and mouse models to evaluate safety, efficacy, and duration of effect. Molecular and physiological readouts include delivery of rAAV genome, *ST3GAL5* expression, restoration of gangliosides, body and brain weight, motor functions, and survival. For each experiment, sample size reflected the number of independent biological replicates and, here, is provided in the figure legends. Mice were assigned randomly to the experimental or control groups. Data from all animals were included in the analysis with no excluded outlier.

### HeLa cell culture and transfection

HeLa cells were maintained in DMEM, GlutaMAX Supplement (Gibco, catalog 10569-010), supplemented with 10% (vol/vol) FBS (Sigma, catalog F2442) and the antibiotics penicillin and streptomycin (100 U/mL) (Gibco, catalog 15140-122) at 37^o^C with 5% CO_2_. HeLa cells were transfected with Lipofectamine 3000 Transfection Reagent (Invitrogen, catalog L3000015).

### iPSC culture and differentiation

iPSCs from patients with GM3SD were shared by Michael Tiemeyer in the Department of Biochemistry and Molecular Biology, University of Georgia, Athens, GA. iPSCs were maintained in mTESR1 (STEMCELL Technologies, catalog 85850), cultured in plates precoated with Matrigel (Corning, catalog 354277), and passaged with Rho kinase inhibitor (Abcam, catalog Ab120129). The cortical neuron differentiation was described by Shi et al. (52). Briefly, iPSCs were cultured in neural maintenance media (DMEM:F12 + GlutaMax, Thermo Fisher Scientific, catalog 10565018; and Neurobasal, Thermo Fisher Scientific, catalog 21103049) and first induced by neural induction media containing SB431542 (Tocris, catalog 1614) and dorsomorphin (Tocris, catalog 3093) for 12 days to form the neuro-epithelial sheet. Then cells were passaged with dispase (Thermo Fisher Scientific, catalog 17105041) to wells coated with laminin (Sigma-Aldrich, catalog L2020) in neural maintenance medium. Cells were passaged and plated until after differentiation day 35 in the final plates precoated with poly-l-lysine (Sigma-Aldrich, catalog P5899). Neurons were infected with lentiviral vectors in the presence of 8 μg/mL polybrene (Sigma-Aldrich, catalog TR-1003-G).

### Lentiviral vectors

Human *ST3GAL5* cDNA isoforms driven by CMV-enhancer/chicken β-actin promoter were cloned into the lentiviral transfer plasmid pLenti-CSCGW2. The third-generation system was used to package lentiviral vectors (53). Lentivirus vector plasmid was cotransfected with packaging genome plasmids (pMDLg/Prre and pRSV/REV) and envelope plasmid (pHCMV/VSVG) to HEK293T cells using the CaCl_2_ method (54). Lentivirus vector supernatants were harvested at 48 and 72 hours after transfection and high-titer virus was concentrated via ultra-centrifugation. Virus titer was determined using QuickTiter Lentivirus Titer Kit (Cell Biolabs, Inc., catalog VPK-107).

### Western blot

Cell culture was lysed in ice-cold RIPA Lysis and Extraction Buffer (Thermo Fisher Scientific, catalog 89901) with complete, EDTA-free protease inhibitor cocktail (Roche, catalog 4693159001). Cell lysate was then sonicated. Debris was removed by centrifugation (10 minutes, 16,000*g*, 4^o^C) and supernatant was collected. Total protein concentration was determined using a bicinchoninic acid protein assay kit (Thermo Fisher Scientific, catalog 23252). Lysates containing equal amounts of total protein were boiled in Tris-Glycine SDS Sample Buffer (Invitrogen, catalog LC2676) at 95^o^C for 5 min. Primary Abs rabbit anti-ST3GAL5 (Thermo Fisher Scientific, catalog PA5-25730; 1:1000 dilution) and mouse anti-actin (Abcam, catalog ab8226; 1:5000 dilution) and secondary Abs IRDye 680RD donkey anti–rabbit IgG (LI-COR Biosciences, catalog 926-68073; 1:5000 dilution) and IRDye 800CW donkey anti–mouse IgG (LI-COR Biosciences, catalog 926-32212; 1:5000 dilution) were applied to Western blot. Membrane was scanned with a LI-COR Odyssey scanner.

### Immunofluorescence staining

Immunofluorescence (IF) staining was applied to iPSC-derived cortical neurons and mouse brain sections. Cortical neurons were fixed with 4% paraformaldehyde (Electron Microscopy Sciences, catalog 15710) after washing with Dulbecco’s PBS (Thermo Fisher Scientific, catalog 14190144). Following that, cells were permeabilized with 0.2% (vol/vol) Triton X-100 for neural markers, or not, for gangliosides staining and blocked with 5% goat serum (Invitrogen, catalog 50062Z) in 0.2% (vol/vol) Triton X-100. Mouse brains were fixed in 4% paraformaldehyde at 4^o^C overnight. The next day, brains were soaked in 30% sucrose at 4^o^C overnight until balanced. Brains were then mounted in OCT compound (Midland Scientific, catalog Sakura 4583) and stored at –80^o^C until cryosectioning. Brain slices were permeabilized with 0.5% (vol/vol) Triton X-100 and blocked with 5% goat serum (Invitrogen, catalog 50062Z). Primary Abs, chicken anti–microtubule-associated protein 2 (Abcam, catalog ab5392; 1:1000 dilution), mouse anti–β III tubulin (Tuj1) (Abcam, catalog ab78078; 1:1000 dilution), rat anti–COUP-IF-interacting protein 2 (Ctip2) (Abcam, catalog ab18465; 1:500 dilution), rabbit anti–T-box brain transcription factor 1 (Abcam, catalog ab31940; 1:1000 dilution), mouse anti-ganglioside GD1a (DSHB, catalog GD1a-1; 1:100 dilution), mouse anti-ganglioside GD1b (DSHB, catalog GD1b01; 1:100 dilution), mouse anti-ganglioside GT1b (DSHB, catalog GT1b-1; 1:100 dilution), and rabbit anti-GFP (Thermo Fisher Scientific, catalog A-11122) were used in immunodetection in blocking buffer at 4^o^C overnight.

Secondary Abs goat anti–chicken IgY H&L, Alexa Fluor 488 (Abcam, catalog ab150169; 1:1000 dilution), donkey anti–mouse IgG H&L, Alexa Fluor 594 (Abcam, catalog ab150108; 1:1000 dilution), goat anti–rat IgG H&L, Alexa Fluor 647 (Abcam, catalog ab150167; 1:1000 dilution), goat anti–rabbit IgG H&L, Alexa Fluor 488 (Abcam, catalog ab150077; 1:1000 dilution), and goat anti–mouse IgG H&L, Alexa Fluor 488 (Thermo Fisher Scientific, catalog A11029) were incubated within blocking buffer at room temperature for 1 hour. Sections were mounted using Prolong Diamond Antifade Mountant with DAPI (Thermo Fisher Scientific, catalog P36962). Images were taken on a Leica TCS SP8 confocal microscope. Quantification of GD1a and GD1b was performed using the ImageJ software (NIH).

### AAV vectors

Human *ST3GAL5* cDNA isoforms driven by CMV-enhancer/chicken β-actin promoter and human *ST3GAL5* cDNA isoforms plus miR122 binding sites driven by Syn1 promoter were cloned into AAV plasmids. The plasmids were sequenced throughout the expression cassette, and the integrity of ITRs was confirmed by restriction enzyme digestion. AAV vectors were produced by transient triple transfection in HEK293 cells and purified by CsCl gradient sedimentation for AAV9 or by iodixanol gradient sedimentation for PHP.eB vectors. Vector titers were determined by droplet digital PCR (ddPCR), and vector purity was assessed by gel electrophoresis followed by silver staining.

### Vector DNA extraction and alkaline gel electrophoresis

Extractions of vector DNA from 8 × 10^11^ to 1 × 10^13^ gcs were performed by phenol/chloroform and ethanol precipitation, as described previously (55). Vector DNA was subjected to alkaline gel electrophoresis stained with ethidium bromide.

### Animal use

St3gal5^–/–^/B4galnt1^+/–^ male mice were imported from Regeneron Pharmaceuticals, Inc., and bred with C57BL6NTac female mice (Taconic, B6-F). Newborns were genotyped on the date of birth. Briefly, 1 mm was cut from tail tips. Genomic DNA was extracted by boiling in 25 mM NaOH plus 0.4 mM EDTA (pH 8.0) at 100^o^C for 90 minutes, followed by mixing with 40 mM Tris-HCl (pH 8.0). *St3gal5* and *B4galnt1* genes were determined by qPCR using Taqman reagents targeting *St3gal5* (Thermo Fisher Scientific, assay ID: APH6DZ6, 9057mTGU; assay ID: APMFZ6Z, 9057mTGD), *B4galnt1* (LGC Biosearch Technologies, catalog DLOM-RFB-5, assay ID: 15582TU; assay ID: LacZ) and *Tfrc* (Thermo Fisher Scientific, catalog 4458367). Primer and probe sequences can be found in Supplemental Table 1. To harvest tissues, mice were anesthetized with isoflurane and transcardially perfused with ice-cold PBS. Tissues were immediately dissected, snap-frozen in liquid nitrogen, and stored at –80^o^C. Facial-vein injections were performed on P1 via the right facial vein at 100 μL of 3 × 10^11^ gcs per pup. ICV injections were performed on P1 at 4 μL of 3 × 10^10^ GC bilaterally per pup or P4 at 4 μL of 6 × 10^10^ GC bilaterally per pup. After the procedure, pups were cleaned with 70% ethanol and rubbed with bedding material.

### DNA/RNA extraction, quantitative real-time PCR, and ddPCR

Total DNA and RNA were extracted from snap-frozen mouse tissues using the AllPrep DNA/RNA Mini kit (Qiagen, catalog 80204). The viral vector gc number was determined in a multiplexed reaction using ddPCR Supermix for Probes (No dUTP) (Bio-Rad, catalog 1863024) and Taqman reagents targeting *ST3GAL5* (Thermo Fisher Scientific, Assay ID: APGZHGD) and *Tfrc* (Thermo Fisher Scientific, catalog 4458367). Total RNA (1 μg) was reverse transcribed into cDNA using the High-Capacity cDNA Reverse Transcription Kit (Applied Biosystems, catalog 4368813). Exogenous human *ST3GAL5* and mouse *St3gal5* cDNA were quantified in a multiplexed reaction using Taqman reagents targeting *ST3GAL5* (Thermo Fisher Scientific, Assay ID: APGZHGD), *St3gal5* (Thermo Fisher Scientific, Assay ID: Mm00488232_m1), and *Gusb* (Thermo Fisher Scientific, Assay ID: Mm01197698_m1). ddPCR was performed with a QX200 ddPCR system (Bio-Rad). Quantitative real-time PCR was performed on a ViiA 7 Real-Time PCR system using Taqman gene expression master mix (Thermo Fisher Scientific, catalog 4369016) and Taqman reagents targeting *Chop* (Thermo Fisher Scientific; Assay ID: Mm01135937_g1) and *Tnfa* (Thermo Fisher Scientific; Assay ID: Mm00443260_g1).

### Mass spectrometry

The sample preparation and analysis were described by Fan et al. (56). Briefly, brain tissue samples were homogenized in water (4 mL/g wet tissue) using an Omni Bead Ruptor (Cole-Parmer, catalog Mfr19-628). The LacCer, GM1, GM2, and GM3 were extracted from 50 μL of homogenate or serum with 200 μL of methanol containing d3-Lc (16:0) (Matreya LLC, catalog 1534), d3-GM1 (18:0) (Matreya LLC, catalog 2050), d3-GM2 (18:0) (Matreya LLC, catalog 2051), and d3-GM3 (18:0) (Matreya LLC, catalog 2052) as the internal standards for LacCer, GM1, GM2, and GM3, respectively. Quality control samples were prepared by pooling aliquots of positive samples, and every 5 study samples were injected to monitor instrument performance throughout these analyses.

The analysis of LacCer, GM1, GM2, and GM3 was performed on a Shimadzu 20AD HPLC system and a SIL-20AC autosampler coupled to a 6500QTRAP+ mass spectrometer (AB Sciex) operated in positive multiple-reaction monitoring mode. Data processing was conducted with Analyst 1.6.3 (Applied Biosystems). The relative quantification data for all analytes are presented as the peak ratios of analytes to their internal standard.

### Mouse monitoring and behavioral assays

Mice were blindly weighed every other day until weaning at 21 days old. After weaning, each mouse was weighed and evaluated for adverse events weekly by a trained observer.

#### Negative geotaxis.

A negative geotaxis assay was conducted every other day for P9–P15 pups on a 45^o^ inclined plane. Prior to the test, animals were placed on the plane to acclimate for 1 minute. The mouse head was facing downward; success was marked when the mouse rotated 180^o^ to the head-up position, and failure was when the mouse dropped off the plane. Whether the mouse could finish the assay was recorded. Each mouse was tested 3 times and the success rate of completing the assay was plotted.

#### Accelerated rotarod.

Coordinated motor functions were examined in treated mice and littermates using the 4–40 rpm accelerating rotarod test. Mice were tested at 6 and 10 weeks old. Tested mice were trained 2 days before the testing day. Prior to the test, the animals were placed on the rotarod machine to acclimate for at least 1 minute. Each mouse was tested 3 times and the best latency to fall was recorded and plotted.

### Histology and immunohistochemistry

Mouse brain and liver were fixed in 10% formalin (Thermo Fisher Scientific, catalog SF100-20). Paraffin embedding, sectioning, H&E staining, TUNEL staining (Roche, catalog 11684817910), and immunohistochemistry (IHC) were performed by the Morphology Core at University of Massachusetts Chan Medical School under standard conditions. Mouse anti–GFAP Ab (EMD Millipore, catalog MAB360; 1:500 dilution) was used in IHC. Images were taken on a Leica DM5500 B microscope. The quantification of GFAP IHC was performed using the Image FIJI software as previously described (57).

### ProcartaPlex multiplex immunoassays

Total protein was extracted in ice-cold RIPA Lysis and Extraction Buffer (Thermo Fisher Scientific, catalog 89901) with complete, EDTA-free protease inhibitor cocktail (Roche, catalog 4693159001) from snap-frozen tissues. Protein concentration was determined using a bicinchoninic acid protein assay kit (Thermo Fisher Scientific, catalog 23252). Normalized protein extracts were loaded on a ProcartaPlex Mix & Match panel (Thermo Fisher Scientific). Values were acquired by Bio-Plex MAGPIX (Bio-Rad).

### mRNA-Seq

RNA-Seq was carried out by Novogene under standard conditions. RNA was extracted using the Trizol phase separation method from cell debris. Isolated RNA sample integrity and concentration were assessed by the Agilent Bioanalyzer 2100. RNA (1 μg/sample) was used as input material for RNA sample preparations. Sequencing libraries were generated using NEBNext Ultra RNA Library Prep Kit for Illumina (New England BioLabs, catalog E7770L) following manufacturer’s recommendations. Briefly, mRNA was purified from total RNA using poly-T oligo-attached magnetic beads. Fragmentation was carried out using divalent cations under elevated temperature in NEBNext First Strand Synthesis Reaction Buffer (5×) (New England BioLabs). First-strand cDNA was synthesized using a random hexamer primer and M-MuL V Reverse Transcriptase (RNase H-). Second-strand cDNA synthesis was subsequently performed using DNA Polymerase I and RNase H. Final library quantities were assessed by the Agilent Bioanalyzer 2100 system. The clustering of the index-coded samples was performed on a cBot Cluster Generation System using PEE Cluster Kit cBot-HS (Illumina) according to the manufacturer’s instructions. After cluster generation, the library preparations were sequenced on an Illumina NovaSeq 6000 platform and paired-end reads were generated.

For data analysis, a 3′ adapter sequence was removed using Trimmomatic with the following ILLUMINACLIP parameters: min_length, 10; seed mismatches, 2; palindrome clip threshold, 30; simple clip threshold, 5. Then, reads were mapped to mouse_mm10_gencode_ using STAR. To estimate expression levels, RSEM55 was used to align reads to a predefined set of transcripts from GENCODE. Finally, the RSEM quantification matrix (i.e., estimated counts for each gene and/or for each annotated isoform) was used for differential gene expression analysis. The count matrix was loaded into DEBrowser software for interactive analysis. Data analysis was performed on the RNA-Seq pipeline of the DolphinNext (57).

### Statistics

All data are presented as mean (SD) and were analyzed using GraphPad Prism software (version 9). Two-tailed Student’s *t* test was used to compare 2 groups, and 1-way ANOVA was used to compare data among multiple groups. Animal weight was analyzed by 2-way ANOVA, and survival was analyzed by log-rank (Mantel-Cox) test. In the figures, *P* values are indicated by asterisks, as follows: *P < 0.05, **P < 0.01, ***P < 0.001, ****P < 0.0001.

### Study approval

All animal procedures were reviewed and approved by IACUC at University of Massachusetts Chan Medical School and performed in compliance with all relevant ethical regulations.

### Data and materials availability

mRNA-Seq data can be found in the National Center for Biotechnology Information’s Gene Expression Omnibus (GEO) using GEO series accession number GSE201587. Other data supporting the findings of this study are available within the article or from the corresponding authors upon reasonable request.

## Author contributions

GG and KAS conceived the project; HY, DW, KAS, and GG designed the experimental plan; HY performed cell, animal, and mouse tissue experiments; HY analyzed data with critical input from RHB, DW, and GG; HY, DW, and GG wrote manuscript; KAS and GG supervised project.

## Supplementary Material

Supplemental data

## Figures and Tables

**Table 1:**
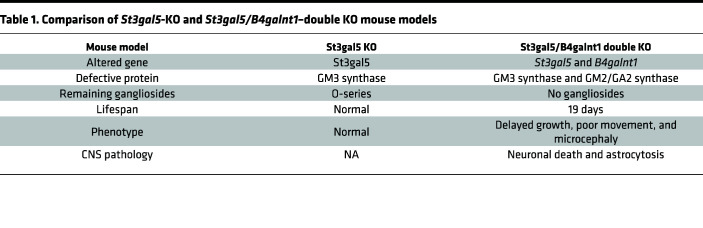
Comparison of *St3gal5*-KO and *St3gal5/B4galnt1*–double KO mouse models

## Version 1 04/04/2023

In-Press Preview

## Version 2 05/08/2023

Electronic publication
