# Supplementary material for: Rescue of GM3 synthase deficiency by spatially controlled, rAAV-mediated ST3GAL5 delivery
Source: JCI Insight. 2023 May 8;8(9):e168688. doi: 10.1172/jci.insight.168688 (PMC10243808; doi:10.1172/jci.insight.168688)
Supplement: Supplemental data [file jciinsight-8-168688-s167.pdf]

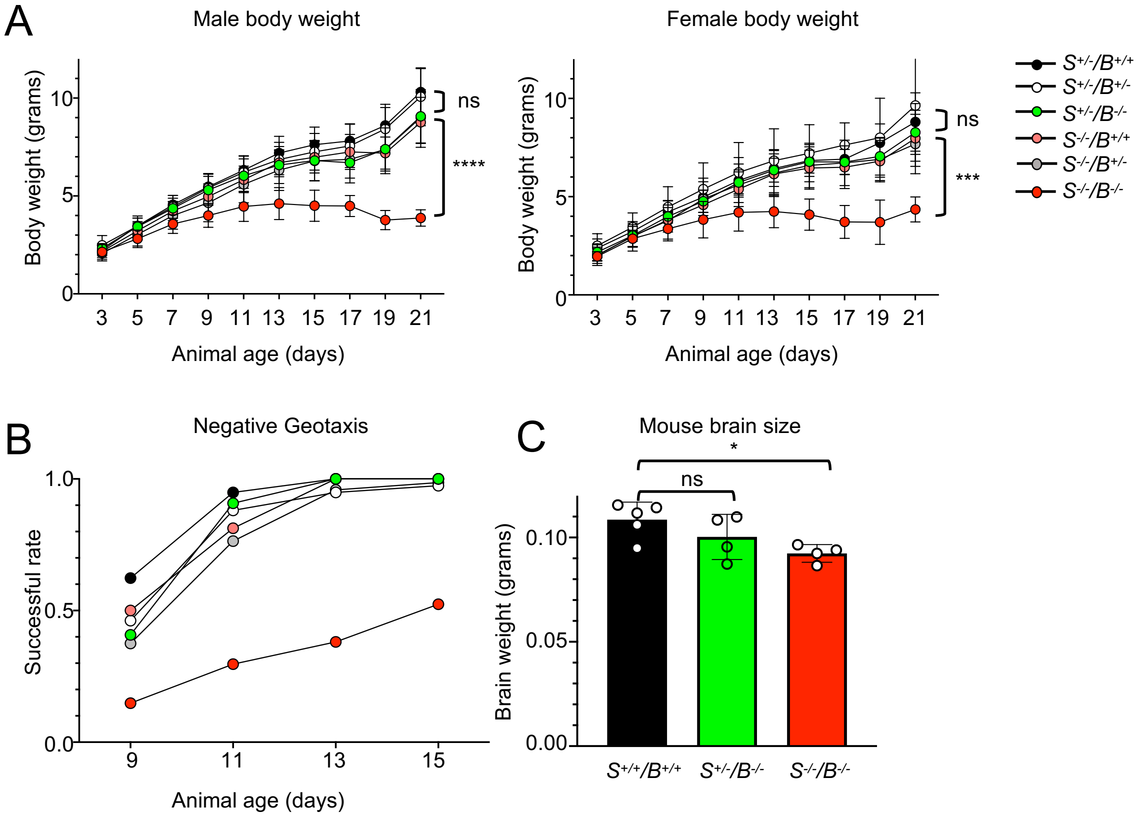

Figure S1. *St3gal5*$^{-/-}$/*B4galnt1*$^{-/-}$ mice exhibit impaired growth and motor function.
(**A**). A time course body weight from postnatal pups aging from 3 days old to 21 days old. Data are mean ± s.d. of 8-10 animals. Statistical analysis was performed by two-way ANOVA, followed by Sidak's multiple comparisons test. ***P < 0.001, ****P < 0.0001, ns: not significant. (**B**). Negative geotaxis successful rate from postnatal pups aging from 9 days old to 15 days old. Data are normalized from 10 animals. (**C**). Quantification of brain weight from wildtype mice or *St3gal*$^{+/-}$/*B4galnt1*$^{-/-}$ mice or *St3gal5*$^{-/-}$/*B4galnt1*$^{-/-}$ mice at 1-day old. Data are mean ± s.d. of 4-5 animals. Statistical analysis was performed by one-way ANOVA, followed by Dunnett's multiple comparisons test. *p<0.05, ns: not significant.

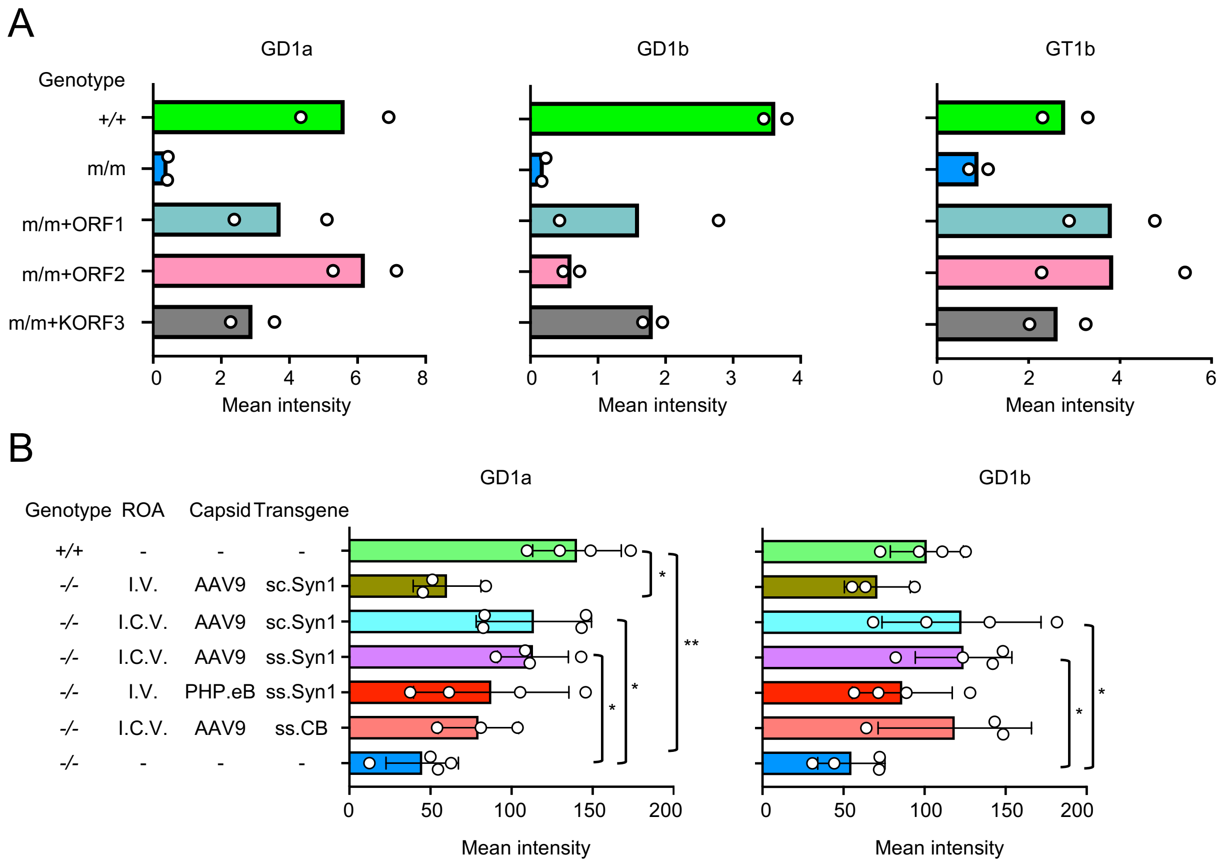

Figure S2. Quantification of gangliosides.
(**A**). Quantification of mean intensity of GD1a or GD1b or GT1b immunofluorescence from cortical neurons by lentiviral vectors expressing *ST3GAL5* ORFs. +/+, wildtype; *m/m*, *ST3GAL5*$^{mut/mut}$. Data are mean of 2 slides per group. (**B**). Quantification of mean intensity of GD1a or GD1b immunofluorescence from cortex of *St3gal5*$^{+/+}$ and *St3gal5*$^{-/-}$ mice, with (+) or without (-) AAV.ST3GAL5 treatments. ROA, route of administration; sc, self-complimentary vector genome; ss, single-strand vector genome; Syn1, Synapsin1 promoter; CB, chicken-beta actin promoter. Data are mean ± s.d. of 3-4 animals per group. Statistical analysis was performed by one-way ANOVA, followed by Sidak's multiple comparisons test. *p<0.05, **p<0.01, ns: not significant.

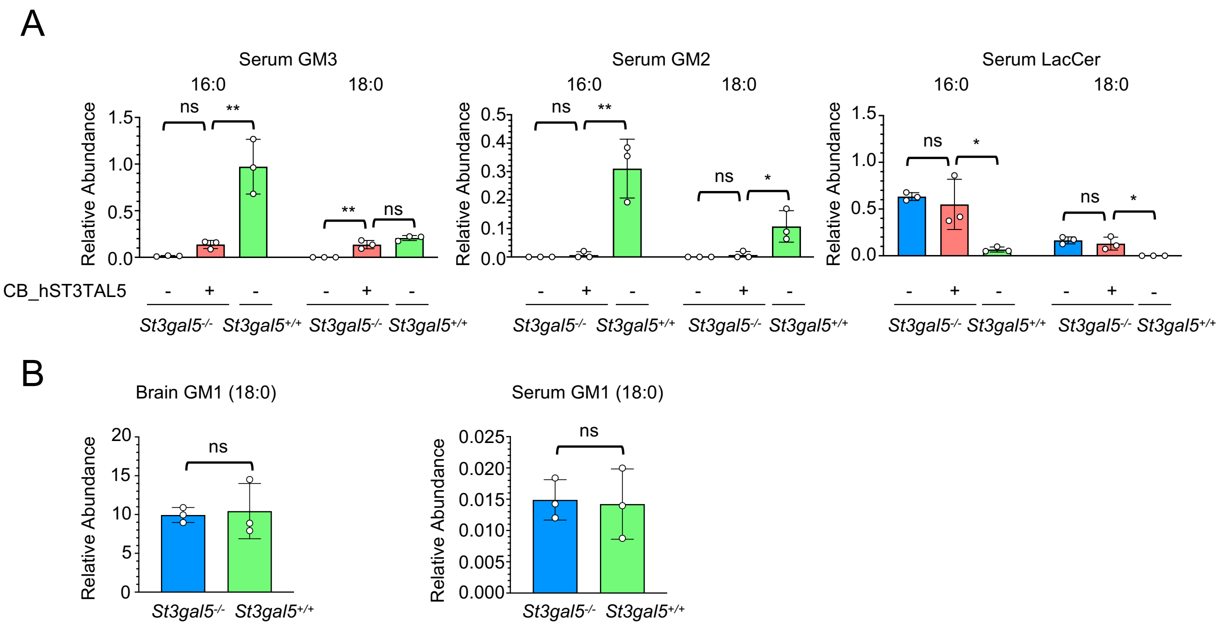

Figure S3. I.C.V. delivery of *ST3GAL5* restores limited peripheral gangliosides production in *St3gal5*$^{-/-}$ mouse model.

(**A**). Mass Spectrometry (MS) quantification of GM3 (16:0, 18:0), GM2 (16:0, 18:0), and LacCer (16:0, 18:0) from serum of *St3gal5*$^{+/+}$ and *St3gal5*$^{-/-}$ mice, with (+) or without (-) rAAV9.CB.hST3GAL5 treatment. Data are mean ± s.d. of 3 animals per group. Statistical analysis was performed by one-way ANOVA, followed by Sidak's multiple comparisons test. *$p<0.05$, **$p<0.01$, ns: not significant. (**B**). Mass Spectrometry (MS) quantification of GM1 (18:0) from brain and serum of *St3gal5*$^{+/+}$ and *St3gal5*$^{-/-}$ mice. Data are mean ± s.d. of 3 animals per group. Statistical analysis was performed by student t-test, ns: not significant.

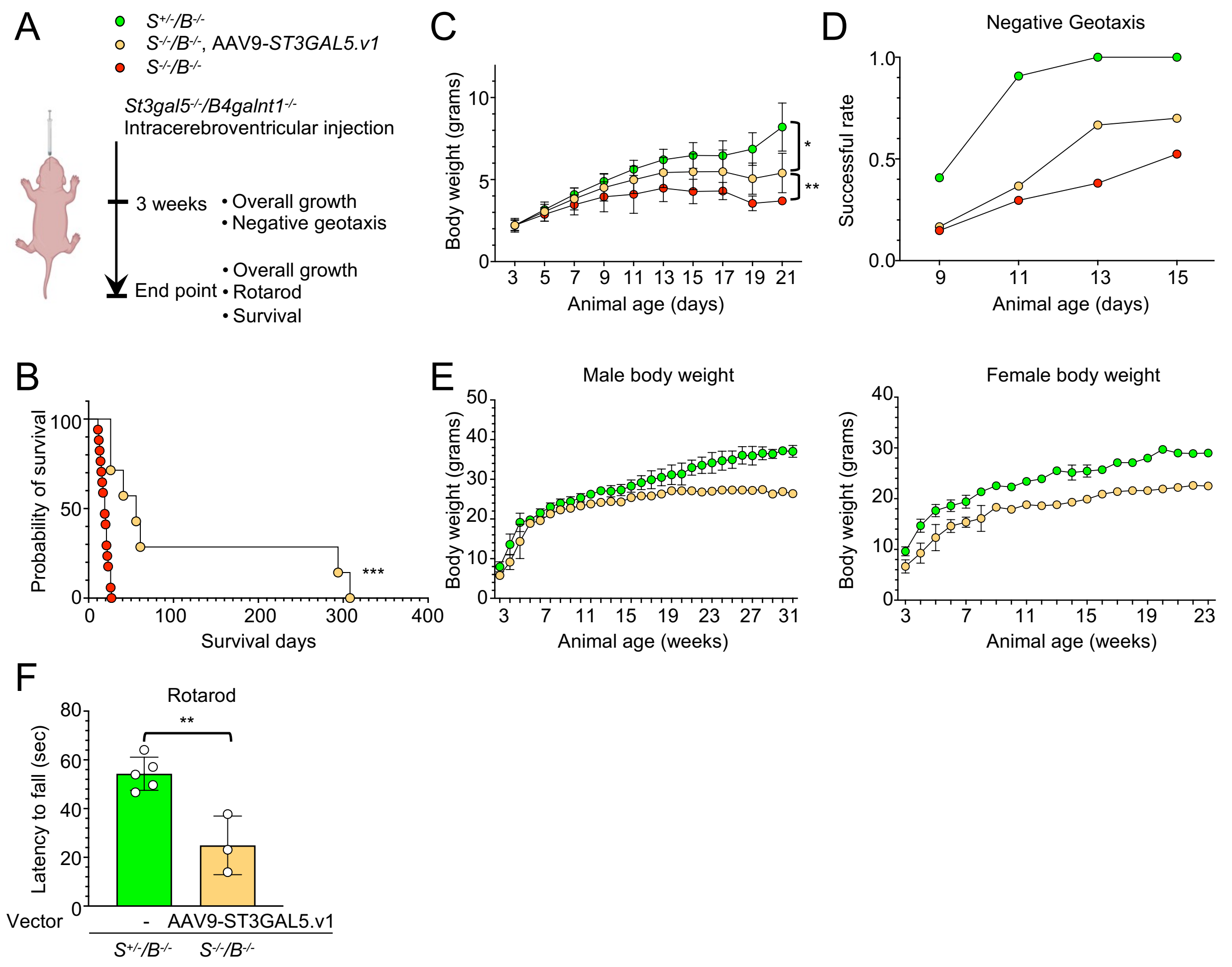

Figure S4. I.C.V. delivery of rAAV9-ST3GAL5.v1 rescues phenotypical changes in *St3gal5*$^{-/-}$/*B4galnt1*$^{-/-}$ mouse model.

(**A**). Schematic of intracerebroventricular (I.C.V.) delivery of rAAV9-ST3GAL5.v1 in *St3gal5*$^{-/-}$/*B4galnt1*$^{-/-}$ mouse model. (**B**). Median survival of *St3gal5*$^{-/-}$/*B4galnt1*$^{-/-}$ mice, with or without rAAV9.CB.hST3GAL5 treatment. Data are plotted as probability of survival from 7-17 animals. Statistical analysis was performed by Lon-rank (Mantel-Cox) test. ***p<0.001. (**C**). A time course body weight from postnatal pups aging from 3 days old to 21 days old. Data are mean ± s.d. of 8-10 animals. Statistical analysis was performed by two-way ANOVA, followed by Sidak's multiple comparisons test. *p<0.05, **p<0.01. (**D**). Negative geotaxis successful rate from postnatal pups aging from 9 days old to 15 days old. Data are normalized from 8-10 animals. (**E**). Body weight of male and female at postweaning stage. Data are represented as mean ± s.d. of 3-4 animals. (**F**). Quantification of rotarod assay for *St3gal5*$^{+/-}$/*B4galnt1*$^{-/-}$ mice and rAAV9-ST3GAL5.v1 treated *St3gal5*$^{-/-}$/*B4galnt1*$^{-/-}$ mice at 6 weeks old. Data are represented as mean ± s.d. of 3-5 animals. Statistical analysis was performed by student t-test. **p<0.01.

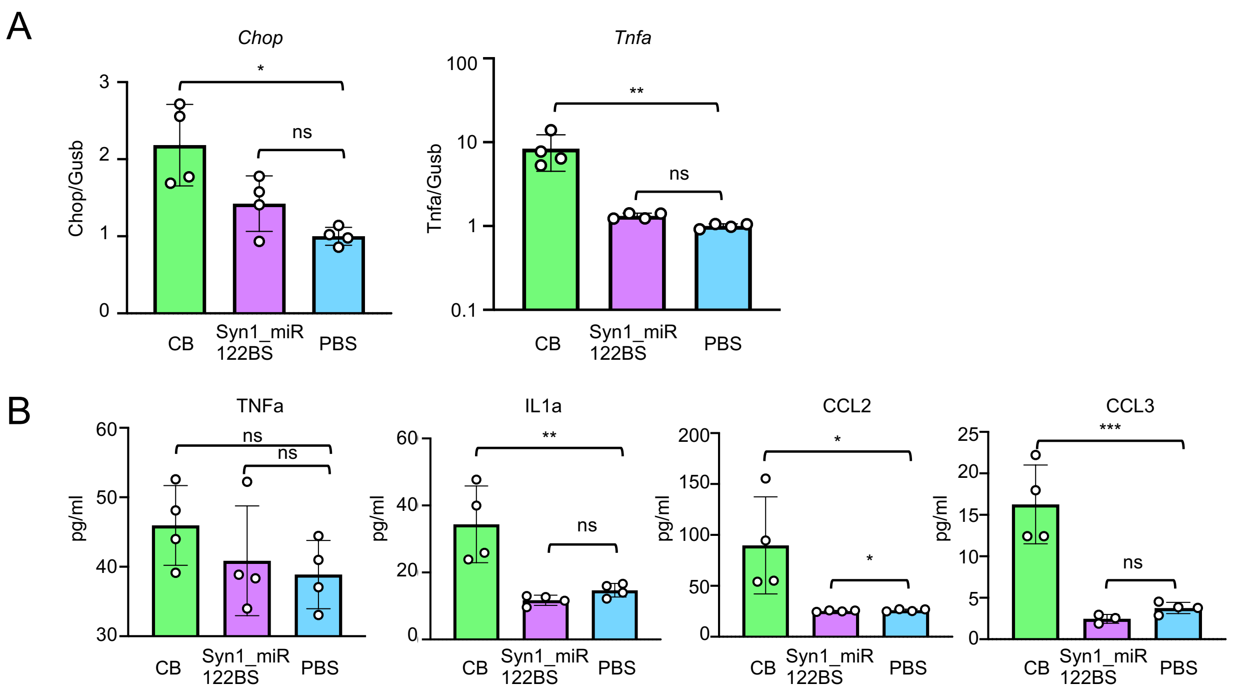

Figure S5. *ST3GAL5* overexpression in liver induces cellular toxicity.
(**A**). qPCR quantification of *Chop* and *Tnfa* mRNA in the liver of wildtype mice with rAAV9.CB.hST3GAL5 or rAAV9.hSyn1.hST3GAL5.miR122BS or PBS treatments. Data are mean ± s.d. of 4 animals per group. Statistical analysis was performed by one-way ANOVA, followed by Sidak's multiple comparisons test. *p<0.05, **p<0.01, ns: not significant. (**C**). Quantification of enzyme-linked immunosorbent assay (ELISA) of TNFA, IL1a, CCL2, and CCL3 protein in the liver of wildtype mice with rAAV9.CB.hST3GAL5 or rAAV9.hSyn1.hST3GAL5.miR122BS or PBS treatments. Data are mean ± s.d. of 4 animals per group. Statistical analysis was performed by one-way ANOVA, followed by Sidak's multiple comparisons test. *p<0.05, **p<0.01, ***p<0.001, ns: not significant.

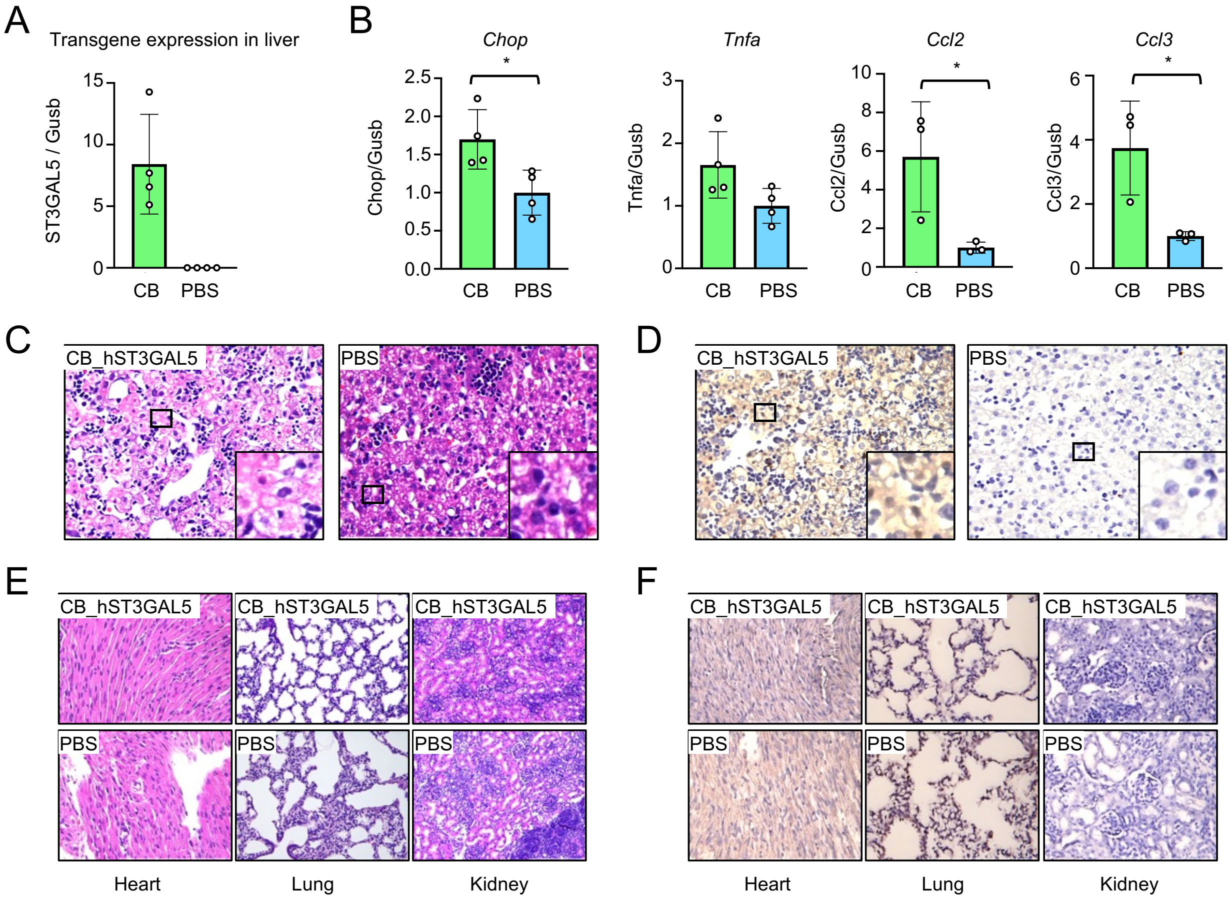

Figure S6. *ST3GAL5* overexpression in liver induces cellular toxicity in *St3gal5*$^{-/-}$ mice. (**A**). ddPCR quantification of *ST3GAL5* mRNA in the liver of *St3gal5*$^{-/-}$ mice with rAAV9.CB.hST3GAL5 or PBS treatments. Data are mean ± s.d. of 4 animals per group. (**B**). ddPCR quantification of *Chop*, *Tnfα*, *CCL2*, and *CCL3* mRNA in the liver of *St3gal5*$^{-/-}$ mice with rAAV9.CB.hST3GAL5 or PBS treatments. Data are mean ± s.d. of 3-4 animals per group. Statistical analysis was performed by student t-test. *p<0.05. (**C**). Representative images of hematoxylin and eosin (H&E) staining of liver sections from *St3gal5*$^{-/-}$ mice injected with rAAV9.CB.hST3GAL5 or PBS. (**D**). Representative images of terminal deoxynucleotidyl transferase dUTP nick end labeling (TUNEL) staining of liver sections from *St3gal5*$^{-/-}$ mice injected with rAAV9.CB.hST3GAL5 or PBS. (**E**). Representative images of hematoxylin and eosin (H&E) staining of heart, lung, and kidney sections from *St3gal5*$^{-/-}$ mice injected with rAAV9.CB.hST3GAL5 or PBS. (**F**). Representative images of terminal deoxynucleotidyl transferase dUTP nick end labeling (TUNEL) staining of heart, lung, and kidney sections from *St3gal5*$^{-/-}$ mice injected with rAAV9.CB.hST3GAL5 or PBS.

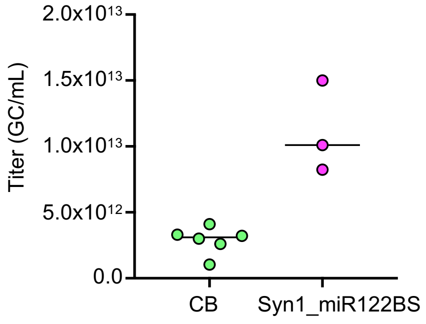

Figure S7. Packaging efficiency of rAAV9.CB.hST3GAL5 and rAAV9.Syn1.hST3GAL5.miR122BS. Quantification of vector titer from AAV9 vector preparation. Data are mean of 3-6 preparations per group.

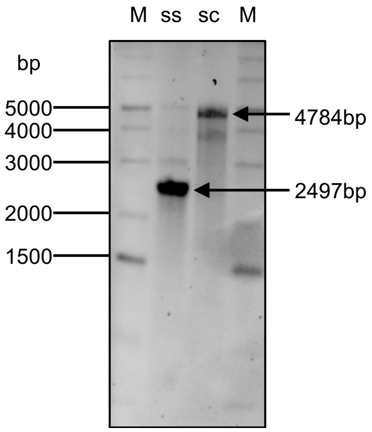

Figure S8. Analysis of genome sizes of ssAAV9.Syn1.hST3GAL5.miR122BS and scAAV9.Syn1.hST3GAL5.miR122BS vectors.
Alkaline gel showing the packaged genome sizes of ssAAV9.Syn1.hST3GAL5.miR122BS and scAAV9.Syn1.hST3GAL5.miR122BS vectors. M: DNA ladder; ss: ssAAV9.Syn1.hST3GAL5.miR122BS, expected genome size is 2,497bp; sc: scAAV9.Syn1.hST3GAL5.miR122BS vectors, expected genome size is 4,784bp; bp: basepair

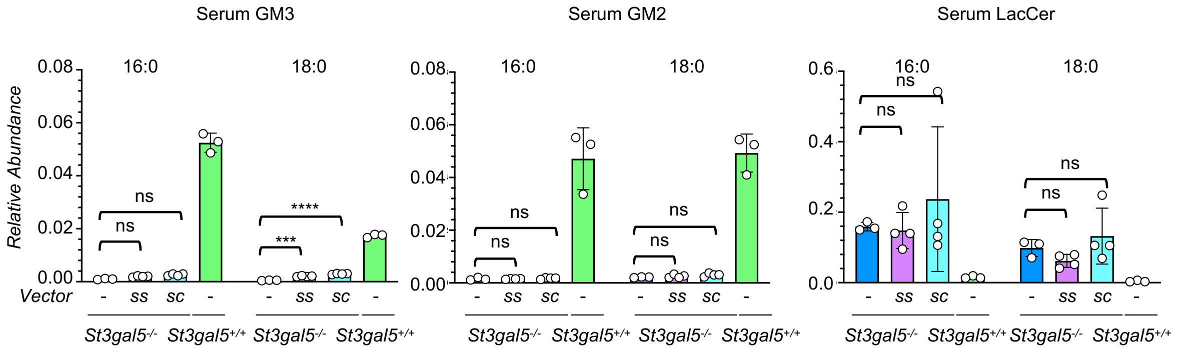

Figure S9. Second generation of *ST3GAL5* replacement vector does not restore peripheral gangliosides production in *St3gal5*$^{-/-}$ mouse model.

Mass Spectrometry (MS) quantification of GM3 (16:0, 18:0), GM2 (16:0, 18:0), and LacCer (16:0, 18:0) from serum of *St3gal5*$^{+/+}$ and *St3gal5*$^{-/-}$ mice, with ssAAV9.ST3GAL5.v2 or scAAV9.ST3GAL5.v2 or no treatment. Data are mean ± s.d. of 3-4 animals per group. Statistical analysis was performed by one-way ANOVA, followed by Sidak's multiple comparisons test. *p<0.05, **p<0.01, ***p<0.001, ****p<0.0001, ns: not significant.

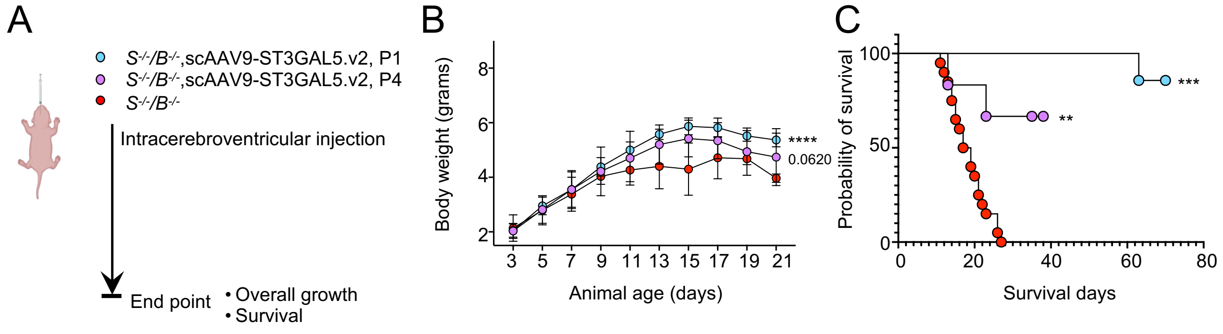

Figure S10. Treatment at a later postnatal stage of scAAV9-ST3GAL5.v2 rescues phenotypical changes in *St3gal5*$^{-/-}$/*B4galnt1*$^{-/-}$ mouse model.

(**A**). Schematic of intracerebroventricular (I.C.V.) delivery of rAAV9-ST3GAL5.v2 in *St3gal5*$^{-/-}$/*B4galnt1*$^{-/-}$ mouse model. (**B**). A time course body weight from postnatal pups aging from 3 days old to 21 days old. Data are mean ± s.d. of 6-10 animals. Statistical analysis was performed by two-way ANOVA, followed by Sidak's multiple comparisons test. ****P < 0.0001. (**C**). Median survival of *St3gal5*$^{-/-}$/*B4galnt1*$^{-/-}$ mice, with or without rAAV9-ST3GAL5.v2 treatment. Data are plotted as probability of survival from 6-17 animals. Statistical analysis was performed by Lon-rank (Mantel-Cox) test. **p<0.01, ****p<0.0001.

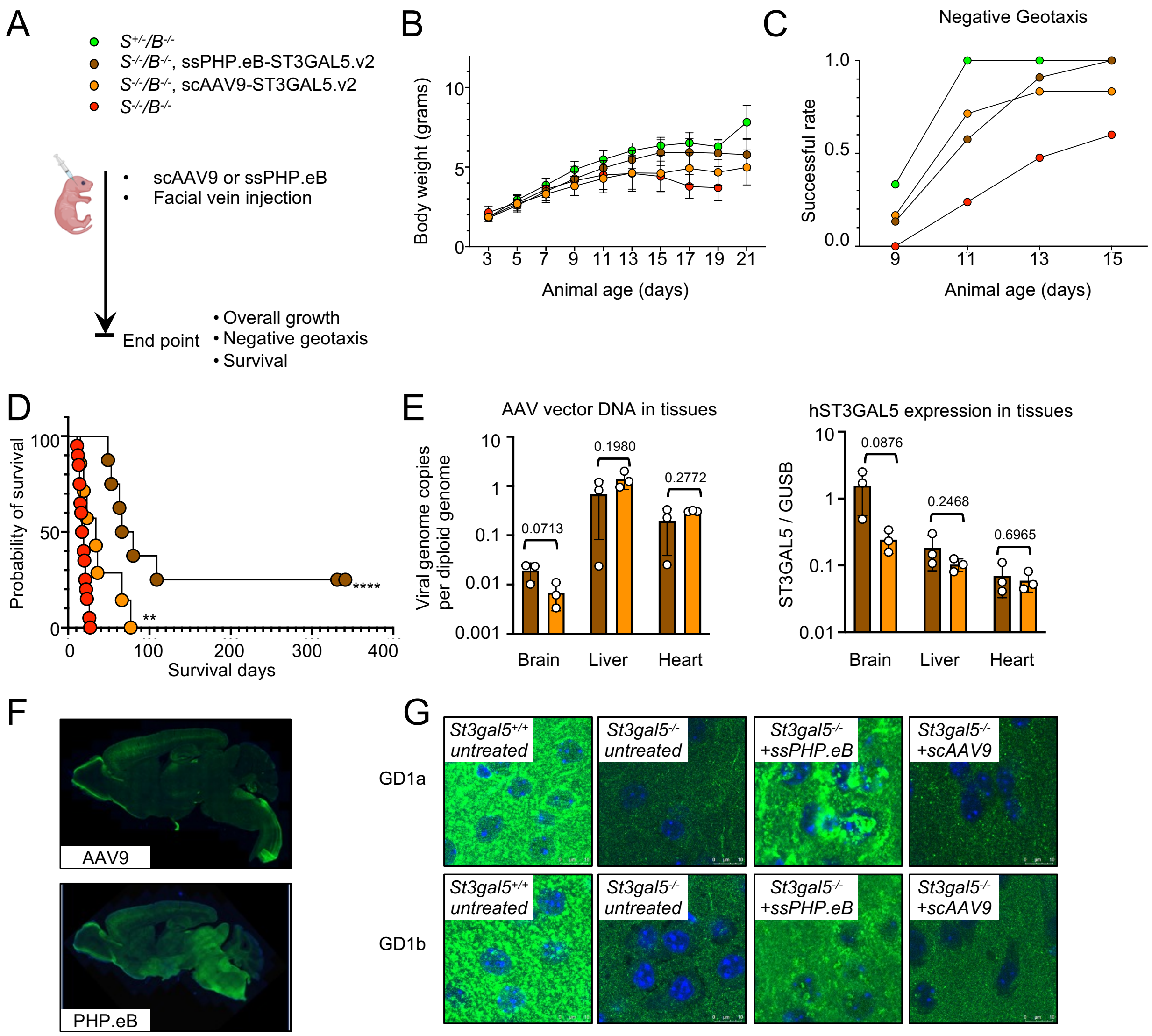

Figure S11. Systematic delivery of second generation *ST3GAL5* vector via AAV PHP.eB rescues GM3SD mouse models.
(**A**). Schematic of intravenous (I.V.) delivery of rAAV-ST3GAL5.v2 in mouse models. (**B**). A time course body weight from postnatal pups aging from 3 days old to 21 days old. Data are mean ± s.d. of 7-10 animals. (**C**). Negative geotaxis successful rate from postnatal pups aging from 9 days old to 15 days old. Data are normalized from 8-10 animals. (**D**). Median survival of *St3gal5*$^{-/-}$/*B4galnt1*$^{-/-}$ mice, with scAAV9.ST3GAL5.v2 or ssPHP.eB.ST3GAL5.v2 or no treatment. Data are plotted as probability of survival from 7-20 animals. Statistical analysis was performed by Lon-rank (Mantel-Cox) test. ***p<0.001. (**E**). ddPCR quantification of human *ST3GAL5* cDNA and AAV genome in the brain, liver, and heart of AAV9.ST3GAL5.v2 or PHP.eB.ST3GAL5.v2 treated mice. Data are mean ± s.d. of 3 animals per group. Statistical analysis was performed by student t-test and P values are marked. (**F**). Representative images of EGFP staining in the brain with AAV9.Syn1.EGFP.miR122BS or PHP.eB.Syn1.EGFP.miR122BS treatment (**G**). Representative images of major brain gangliosides in cortex of *St3gal5*$^{-/-}$ mice with scAAV9.ST3GAL5.v2 or ssPHP.eB.ST3GAL5.v2 treatment. GD1a, GD1b, green; nuclei, counterstaining in blue. Quantification is in **Figure S2.**

Oligo name	Oligo sequence 5'-3'
9057mTGU_probe	FAM-TCCAGAGCCACCTTA–MGB
9057mTGU_Fwd	TGGATGCTCAGTCAAAGCCTTT
9057mTGU_Rev	GGAAGGCGTTCCGGAGAA
9057mTGD_probe	FAM-CTCAGCGGCGGCA-MGB
9057mTGD_Fwd	GAGACCAAGTTCCTCCTGAAGCT
9057mTGD_Rev	GCCGTGTTCCGAGTTCTCA
15582TU_probe	FAM-CAGGTTCGGGCGGTTGACCTCACT-BHQ-1
15582TU_Fwd	AGCCTTCCCTTGCCCTTTCTG
15582TU_Rev	AAACAGCCCTCAGCTCCTCAG
LacZ_probe	FAM-CGATACTGTCGTCGTCCCCTCAAACTG-BHQ-1
LacZ_Fwd	GGAGTGCGATCTTCCTGAGG
LacZ_Rev	CGCATCGTAACCGTGCATC

Supplementary Table 1. Primer and probe sequences
